# The effect of vitamin C on bronchoconstriction and respiratory symptoms caused by exercise: a review and statistical analysis

**DOI:** 10.1186/1710-1492-10-58

**Published:** 2014-11-27

**Authors:** Harri Hemilä

**Affiliations:** Department of Public Health, University of Helsinki, POB 41, Mannerheimintie 172, FIN-00014 Helsinki, Finland

**Keywords:** Anti-asthmatic agents, Ascorbic acid, Cough, Histamine, Exercise-induced asthma, Forced expiratory flow rates, Meta-analysis, Prostaglandin, Randomized controlled trial, The lungs

## Abstract

Physical activity increases oxidative stress and therefore the antioxidant effects of vitamin C administration might become evident in people undertaking vigorous exercise. Vitamin C is involved in the metabolism of histamine, prostaglandins, and cysteinyl leukotrienes, all of which appear to be mediators in the pathogenesis of exercise-induced bronchoconstriction (EIB). Three studies assessing the effect of vitamin C on patients with EIB were subjected to a meta-analysis and revealed that vitamin C reduced postexercise FEV_1_ decline by 48% (95% CI: 33% to 64%). The correlation between postexercise FEV_1_ decline and respiratory symptoms associated with exercise is poor, yet symptoms are the most relevant to patients. Five other studies examined subjects who were under short-term, heavy physical stress and revealed that vitamin C reduced the incidence of respiratory symptoms by 52% (95% CI: 36% to 65%). Another trial reported that vitamin C halved the duration of the respiratory symptoms in male adolescent competitive swimmers. Although FEV_1_ is the standard outcome for assessing EIB, other outcomes may provide additional information. In particular, the mean postexercise decline of FEF_50_ is twice the decline of FEV_1_. Schachter and Schlesinger (1982) reported the effect of vitamin C on exercise-induced FEF_60_ levels in 12 patients suffering from EIB and their data are analyzed in this paper. The postexercise FEF_60_ decline was greater than 60% for five participants and such a dramatic decline indicates that the absolute postexercise FEF_60_ level becomes an important outcome in its own right. Vitamin C increased postexercise FEF_60_ levels by 50% to 150% in those five participants, but had no significant effect in the other seven participants. Thus, future research on the effects of vitamin C on EIB should not be restricted to measuring only FEV_1_. Vitamin C is inexpensive and safe, and further study on those people who have EIB or respiratory symptoms associated with exercise is warranted.

**Electronic supplementary material:**

The online version of this article (doi:10.1186/1710-1492-10-58) contains supplementary material, which is available to authorized users.

## Introduction

Exercise-induced bronchoconstriction (EIB) describes the acute narrowing of the airways that occurs as a result of vigorous exercise [1–3]. The emergence of EIB depends on the kind and level of physical activity, and also on the humidity and temperature of the inhaled air [1–3].

Only about 10% of the non-asthmatic general population suffer from EIB, whereas up to 90% of asthmatics may suffer from EIB [2–4]. Thus EIB is a common phenotype of asthma. EIB is also common among competitive athletes even if they do not have asthma, and it is particularly prevalent in endurance sports, such as running, winter sports and swimming [2, 3, 5].

Usually, a decline of 10% or greater in FEV_1_ after exercise is classified as EIB, but other cut off limits have also been used [1–3]. However, EIB is not an arbitrary dichotomous condition, instead there is a continuous variation in the possible level of FEV_1_ decline such that the 9% and 11% decline levels in FEV_1_ are not biologically different phenomena, although they fall on either side of the usual cut off level. A single constant percentage point cut off limit is thus simplistic. It is more useful to analyze the phenomenon as a continuous variable rather than a dichotomous variable. This issue is relevant when planning appropriate statistical analysis of outcomes related to EIB.

Symptoms are much more important than laboratory values for patients. However, the correlation between the declines in postexercise FEV_1_ values and postexercise respiratory symptoms is poor [4, 6, 7]. Therefore, respiratory symptoms should be recorded concurrently with the pulmonary function tests.

The stimulus for EIB seems to be the loss of water caused by increased ventilation. This leads to the release of mediators such as histamine, prostaglandins and leukotrienes, all of which cause bronchoconstriction [1–3, 8]. Nitric oxide also plays a role in the pathogenesis of EIB [9, 10]. Finally, oxidative stress seems to play role in the emergence of EIB [11, 12].

### Vitamin C: exercise and airways

Physical activity increases oxidative stress [13], and therefore, as an antioxidant vitamin C might have particularly evident effects in people who are participating in vigorous exercise. Electron spin resonance studies have shown that vitamin C administration decreased the levels of free radicals generated during exercise [14, 15] and vitamin C administration attenuated the increases in oxidative stress markers caused by exercise [16–18].

The level of vitamin C in the lungs is high [19], and vitamin C levels in alveolar macrophages and alveolar type II cells are 30 times higher than in plasma [20]. About 10% of vitamin C in the lungs of rats is in a lavageable form [21], but the level of vitamin C in the bronchoalveolar lavage seems to be lower in humans than in rats [22]. In any case, high levels of vitamin C in the lungs imply that the vitamin may protect the lungs against oxidative stress.

Unlike guinea pigs and humans, mice and rats are able to synthesize vitamin C, and these species are able to increase their rates of vitamin C synthesis under certain stressful conditions. Ozone exposure in rats and mice significantly increased vitamin C levels in bronchoalveolar lavage fluid [23, 24], which might serve as a protective response to the higher oxidative stress level being encountered. Exposure to ozone and nitrogen dioxide decreased lung vitamin C levels in guinea pigs, which implies that the vitamin was consumed while it protected against the oxidants [25, 26]. Vitamin C administration in guinea pigs decreased mortality caused by ozone exposure [27, 28] and vitamin C deficiency in guinea pigs increased necrotic injury to type II lung cells upon H_2_O_2_ treatment [29]. Exposure to ozone in humans decreased the vitamin C level in the respiratory tract lining fluid [30]. Thus, given that oxidative stress seems to play a role in EIB [11, 12], vitamin C might protect against EIB through non-specific antioxidant effects. Nevertheless, there are also more specific biochemical mechanisms through which vitamin C may influence pulmonary functions.

Histamine is one of the mediators involved in the pathogenesis of EIB [1–3, 8]. It is released from mast cells, which have a high concentration of vitamin C [31]. Furthermore, the release of histamine causes oxidation of vitamin C in the mast cells [32]. In guinea pigs, a deficiency of vitamin C increased histamine levels in their plasma, urine and lungs [33, 34], whereas a high dosage of vitamin C decreased their plasma histamine levels [35]. In vitamin C deficient guinea pigs, a single dose of vitamin C rapidly decreased plasma and urine histamine levels to normal levels [34]. In rats, vitamin C attenuated the increases in histamine levels, which were caused by various stressful conditions including cold and heat stress [36]. Four trials conducted on humans found that the administration of vitamin C significantly decreased plasma histamine levels [37–40]. Vitamin C decreased bronchoconstriction caused by histamine in living guinea pigs [33, 41–44], and it decreased contractions caused by histamine in isolated guinea pig trachea smooth muscle [45, 46]. Finally, in guinea pigs exposed to ozone, vitamin C decreased bronchial reactivity to histamine [47].

Prostaglandins (PGs) and leukotrienes (LTs) also participate in the pathogenesis of EIB [1–3, 8]. Vitamin C deficiency in guinea pigs increased the level of bronchoconstrictor PGF_2α_ in the trachea [44, 48], and increased the *in vitro* synthesis of PGF_2α_ in lung microsomes [49]. Vitamin C deficiency decreased the production of PGE_2_ in guinea pig trachea [48]; PGE_2_ causes smooth muscle relaxation and may protect against EIB [1, 2]. Furthermore, hyper-responsiveness to histamine in vitamin C deficient guinea pigs was further increased by indomethacin [44], and the relaxing effects of vitamin C on isolated guinea pig trachea were inhibited by indomethacin [46]. Indomethacin also blocked the effect of vitamin C on methacholine-induced bronchoconstriction in humans [50]. The influence of indomethacin on vitamin C effects is a further indication that the pulmonary effects of vitamin C may be partly mediated through the influences of vitamin C on the PG metabolism. Furthermore, vitamin C decreased contractions caused by PGF_2α_ in guinea pig tracheal tube preparations [48]. Finally, the administration of vitamin C in humans reduced the postexercise increase in the urinary markers of bronchoconstrictors PGD_2_ and cysteinyl LTs [51].

Nitric oxide (NO) has also been implicated in the pathogenesis of EIB [9, 10]. The metabolism of NO is altered in EIB patients but it is not correlated with exercise-induced changes in spirometry [9]. Vitamin C was reported to decrease the NO level in EIB patients [51].

A single oral dose of vitamin C can rapidly elevate mucosal vitamin C levels. Nasal lavage fluid vitamin C levels in human subjects increased by three-fold in two hours after a single dose of 1 or 2 g of vitamin C [52, 53]. The rapid transport of ingested vitamin C to the respiratory tract lining fluid implies that even single doses of vitamin C might be effective in protecting against acute increases in oxidative stress in the airways.

### FEV_1_ decline caused by exercise

**Figure 1:**
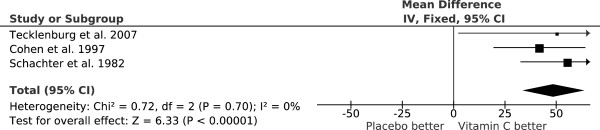
**Reduction of postexercise FEV**_**1**_ **decline by vitamin C.** The vertical lines indicate the 95% CI for the three trials that studied the effect of vitamin C administration on EIB and the squares in the middle of the lines indicate the point estimates of the studies. An effect of 100% would indicate full prevention of postexercise FEV_1_ decline. The diamond shape at the foot indicates the 95% CI for the pooled vitamin C effect: 48% (95% CI: 33% to 64%) reduction in postexercise FEV_1_ decline. For example, Tecklenburg et al. reported a 12.9% FEV_1_ decline after placebo, but only a 6.4% decline after the vitamin C period, which corresponds to a 50% reduction in FEV_1_ decline by vitamin C [51]. This figure is based on data published in [54].

Three randomized, double-blind, placebo-controlled cross-over trials examined the effect of vitamin C (0.5 to 2 g/day) on exercise-induced FEV_1_ decline (Table 1). The pooled effect of vitamin C (Figure 1) indicates a reduction in the postexercise FEV_1_ decline of 48% (95% CI: 33% to 64%) [54, 55]. In one study, Tecklenburg et al. reported that the postexercise FEV_1_ decline was 12.9% after the placebo period, but only 6.4% after a 2-week vitamin C administration, which corresponds to 50% reduction in the postexercise FEV_1_ decline [51] (Figure 1). In other two studies, vitamin C was administered as a single dose 1 or 1.5 hours before the exercise test [56, 57], yet the effects were the same as for the first study (Figure 1). Thus, a single dose of vitamin C before an exercise session appears to be sufficient to generate the same benefit as a 2-week supplementation regime. This may be explained by the rapid transfer of vitamin C to the airway lining fluids [52, 53].

**Table 1:** **Trials on vitamin C and EIB**

Study [ref.]		Characteristics of participants
Schachter & Schlesinger [56]	Selection:	12 subjects with asthma, selected from among employees of Yale University in the USA: “all 12 subjects gave a characteristic description of EIB.” All included participants had at least 20% reduction in FEF_60_ or FEF_60(P)_ after exercise.
	Sex, age:	5 Males, 7 Females; mean age 26 yr (SD 5 yr).
	Exercise test:	All subjects performed the exercise studies on a cycloergometer. Cardiac frequency was measured with an electrocardiograph. Baseline heart rate was obtained and exercise was begun at a constant speed of 20 km/h against zero workload. At the end of each one min interval cardiac frequency was measured and the workload was increased by 150 kilopondmeters per min, keeping pedalling speed constant throughout the experiment. Exercise against progressively larger workloads was continued until either the heart rate reached 170 beats per min or the subject fatigued. Pulmonary function was assessed post-exercise at 0 and 5 min.
Cohen et al. [57]	Selection:	20 patients with asthma in Israel. All of them demonstrated EIB by having a “decline of at least 15%” in FEV_1_ after a standard exercise test.
	Sex, age:	13 Males, 7 Females; mean age 14 yr (range 7 to 28 yr).
	Exercise test:	A 7-min exercise session using the treadmill. Each subject exercised to submaximal effort at a speed and slope to provide 80% of the motional oxygen consumption as adjudged by a pulse oximeter. Pulmonary function was assessed after an 8-min rest.
Tecklenburg et al. [51]	Selection:	8 subjects with asthma from a population of university students and the local community, Indiana USA. All subjects had “documented EIB as indicated by a drop of greater than 10%” in postexercise FEV_1_. “All subjects had a history of chest tightness, shortness of breath and intermittent wheezing following exercise.”
	Sex, age:	2 Males, 6 Females; mean age 24.5 yr (SD 5 yr).
	Exercise test:	Each subject ran on a motorized treadmill which was elevated 1% per min until 85% of age predicted maximum heart rate and ventilation exceeding 40–60% of predicted maximal voluntary ventilation. Subjects maintained this exercise intensity for 6 min. Following the 6-min steady state exercise, the grade of the treadmill continued to increase at 1% per min until volitional exhaustion. Pulmonary function was assessed post-exercise at 1, 5, 10, 15, 20, and 30 min. The maximum percentage fall in FEV_1_ from the baseline (pre-exercise) value was calculated and used as the outcome.

The three EIB trials included a total of only 40 participants. However, the trials were carried out over three different decades and on two different continents. The criteria for EIB differed and the mean ages of the participants were 14 years in one study [57] but 25 and 26 years in the two other studies [51, 56]. Nevertheless, all the studies are consistent with vitamin C halving the postexercise FEV_1_ decline (Figure 1). It is not clear how far this estimate can be generalized, but similar findings from such dissimilar studies indicate that vitamin C may be effective for a wider population who suffer from EIB.

A fourth randomized cross-over trial on 8 participants who suffered from EIB found that the combination of 0.5 g/day of vitamin C along with vitamin E significantly decreased the FEV_1_ decline at 5, 15 and 30 min after exercise [58]. Although this finding is not specific to vitamin C, it is consistent with the benefits of antioxidants. In 5 other participants who did not suffer from EIB, postexercise FEV_1_ decline was not influenced by the combination of the vitamins [58]. This study was published only as an abstract.

### FEF_60_ and PEF declines caused by exercise

FEV_1_ is the standard outcome for assessing whether a patient suffers from EIB [1–3]. However, exercise-induced decline in FEF_25-75_ is twice as great as the decline in FEV_1_, and therefore, FEF_25-75_ may provide relevant information in addition to FEV_1_ data [6, 7, 9, 10, 59–63]. Furthermore, FEF_50_ values essentially give the same information as FEF_25-75_[64].

**Figure 2:**
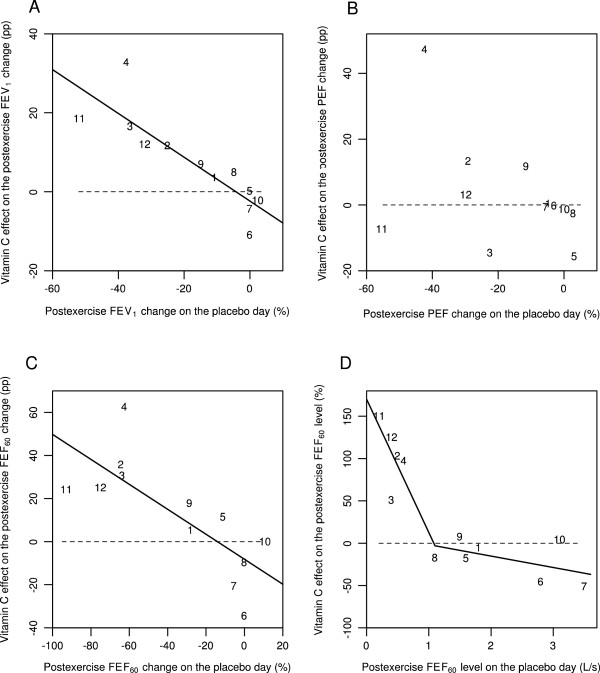
**Effect of vitamin C on 5-min postexercise changes for different pulmonary function outcomes according to the study by Schlesinger and Schachter** [56]**.** Effect of vitamin C on the following 5 min after exercise: **A)** the postexercise FEV_1_ change, **B)** the postexercise PEF change, **C)** the postexercise FEF_60_ change, and **D)** the postexercise FEF_60_ level. Figures 2A, B, and C show the effect of vitamin C in percentage points (pp). For example, on the placebo-day, participant #11 had a postexercise FEV_1_ decline of 52%, and on the vitamin C day a postexercise FEV_1_ decline of 33%, which gives the 19 pp improvement shown in Figure 2A. Figure 2D shows the effect of vitamin C in percentages. For example, on the placebo-day, participant #11 had a postexercise FEF_60_ level of 0.2 L/s, and on the vitamin C day a postexercise FEF_60_ level of 0.5 L/s, which gives the 150% increase shown in Figure 2D. Figure 2 uses the same identification numbers for participants as those used in the original paper [56]. The dash lines indicate equality between vitamin C and placebo. If vitamin C had no effect, the observations would be located randomly and symmetrically around the dash lines. The continuous lines indicate the regression lines. In Figure 2A, the addition of the placebo-day postexercise FEV_1_ change to the model containing the intercept improved the model fit by χ^2^(1 df) =16.5 (P =0.0001). In Figure 2B, the slope did not significantly differ from the null effect and therefore the regression line is not shown. In Figure 2C, addition of the placebo-day postexercise FEF_60_ change to the model containing the intercept improved the model fit by χ^2^(1 df) =10.5 (P =0.001). In Figure 2D, adding the two spline segments with the knot at 1.1 L/s to the model containing only the intercept improved the model fit by χ^2^(2 df) = 24.7 (P = 0.000004). For the statistical methods of Figure 2, see Additional file 1.

Schachter and Schlesinger (1982) studied 12 participants with EIB and published the effects of a single dose of 0.5 g vitamin C on the FEV_1_, PEF, and FEF_60_ levels on each participant before and after the exercise test [56]. Figure 2 shows the data for 5 minutes after the exercise test. As a pulmonary function measure, the FEF_60_ level is close to the FEF_50_ level. On the basis of the slope of the linear regression line (Figure 2A), vitamin C decreased the postexercise FEV_1_ decline by 55% (95% CI: 32% to 78%). The mean decline in postexercise FEV_1_ was 18% on the placebo day. The greatest postexercise FEV_1_ decline on the placebo-day was 52% in participant #11 (Figure 2A).

PEF is not recommended for assessing EIB, since it is less repeatable than FEV_1_[2, 3]. The study by Schachter and Schlesinger found that vitamin C had no consistent effect on postexercise PEF decline (Figure 2B). The linear regression slope did not significantly differ from the null effect, thus it is not shown. However, all of the 5 participants who did benefit of vitamin C as measured by its effects on FEV_1_, also had a substantial postexercise PEF decline of 20% or more on the placebo day. Thus, a high PEF decline on the placebo day identified all the 5 participants who benefited from vitamin C as evaluated by FEV_1_. This finding might have practical importance since PEF measurements are much easier to carry out than spirometry.

The linear regression slope indicated that vitamin C decreased the postexercise FEF_60_ decline by 58% (95% CI: 23% to 92%) (Figure 2C). The mean decline in postexercise FEF_60_ was 35% on the placebo day, which is twice the decline in FEV_1_ (18%). The ratio of about two for the declines in FEF_25-75_ and FEV_1_ has been reported previously [6, 7, 9, 10, 62, 63]. The exercise-induced FEF_60_ decline on the placebo day was greater than 60% in 5 out of the 12 participants, with the greatest decline being 92% for participant #11 (Figure 2C).

A dramatic postexercise FEF_60_ decline in 5 participants indicates that the absolute postexercise level of FEF_60_ becomes an important outcome in its own right. For each of these 5 participants, the postexercise FEF_60_ level was less than 1 L/s on the placebo day (Figure 2D). Moreover, vitamin C administration increased the postexercise FEF_60_ level in these 5 participants by between 50% and 150%. In contrast, no mean difference between the vitamin C and placebo days was detected in the other 7 participants. This indicates that the effect of vitamin C may be restricted to those EIB patients who had postexercise FEF_60_ levels below 1 L/s.

Schachter and Schlesinger also reported the FEF_60(P)_ values, which were based on partial flow volume curves [56]. The effect of vitamin C on FEF_60(P)_ was similar to its effects on FEF_60_. This analysis is shown in Additional file 1.

**Figure 3:**
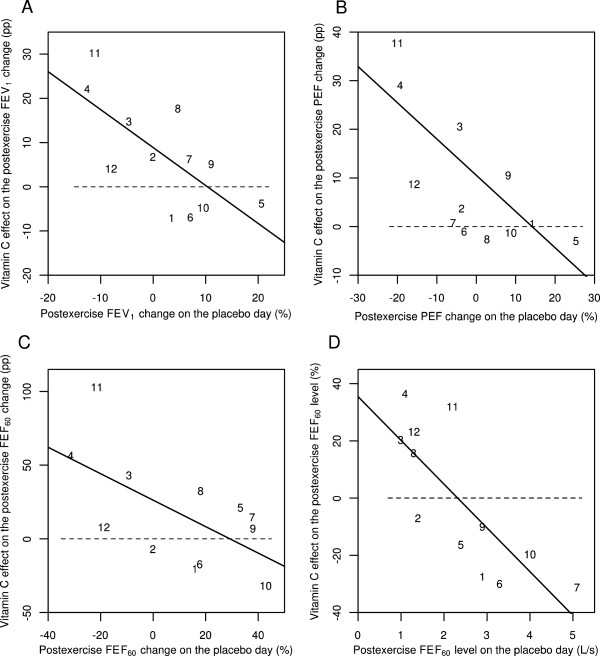
**Effect of vitamin C on 0-min postexercise changes for different pulmonary function outcomes according to the study by Schlesinger and Schachter** [56]**.** Effect of vitamin C on the following immediately (0 min) after exercise: **A)** the postexercise FEV_1_ change, **B)** the postexercise PEF change, **C)** the postexercise FEF_60_ change, and **D)** the postexercise FEF_60_ level. Figures 3A, B, and C show the effect of vitamin C in percentage points (pp). For example, on the placebo-day, participant #11 had a postexercise FEV_1_ change of -11%, and on the vitamin C day a postexercise FEV_1_ change of +19%, which gives the 30 pp improvement shown in Figure 3A. Figure 3D shows the effect of vitamin C in percentages. For example, on the placebo-day, participant #11 had a postexercise FEF_60_ level of 2.2 L/s, and on the vitamin C day a postexercise FEF_60_ level of 2.9 L/s, which gives the 32% increase shown in Figure 3D. Figure 3 uses the same identification numbers for participants as those used in the original paper [56]. The dash lines indicate equality between vitamin C and placebo. If vitamin C had no effect, the observations would be located randomly and symmetrically around the dash lines. The continuous lines indicate the regression lines. In Figure 3A, addition of the placebo-day postexercise FEV_1_ change to the model containing the intercept improved the model fit by χ^2^(1 df) =8.2 (P =0.004). In Figure 3B, addition of the placebo-day postexercise PEF change to the model containing the intercept improved the model fit by χ^2^(1 df) =10.8 (P =0.001). In Figure 3C, addition of the placebo-day postexercise FEF_60_ change to the model containing the intercept improved the model fit by χ^2^(1 df) =5.7 (P =0.02) and in Figure 3D, addition of the placebo-day postexercise FEF_60_ level to the model containing the intercept improved the model fit by χ^2^(1 df) =11.4 (P =0.001). For the statistical methods of Figure 3, see Additional file 2.

Finally, Schachter and Schlesinger reported the FEV_1_, PEF, FEF_60_ and FEF_60(P)_ values also for the time point immediately after the exercise (0 min) [56]. At the 0 minute data, vitamin C and placebo days differ significantly when analyzed by linear regression (Figure 3). On the basis of the slopes, vitamin C decreased the postexercise FEV_1_ changes by 86% (95% CI: 24% to 147%), postexercise PEF changes by 74% (95% CI: 31% to 118%), and postexercise FEF_60_ changes by 90% (95% CI: 9% to 171%). Postexercise FEF_60_ levels were also significantly influenced by vitamin C (P =0.003). Thus, significant effects on postexercise pulmonary function changes by vitamin C can also be seen immediately after the exercise challenge test. This analysis is shown in Additional file 2.

### Respiratory symptoms caused by exercise

In evidence-based medicine (EBM) the primary focus of interest is on clinically relevant outcomes, including symptoms such as cough, sore throat and dyspnea. From the EBM perspective, laboratory outcomes such as FEV_1_ are surrogates and of secondary importance. Postexercise FEV_1_ decline and postexercise respiratory symptoms are poorly correlated [4, 6, 7]. Therefore, the effects of vitamin C administration on respiratory symptoms caused by exercise is an important question that should be concurrently considered along with its effects on the pulmonary function test values. None of the three trials on vitamin C and EIB recorded respiratory symptoms associated with the exercise tests (Table 1). Nevertheless, Tecklenburg et al. did report that asthma symptoms were less intense after the vitamin C administration period compared with the placebo period [51].

Five separate randomized placebo-controlled trials reported the effects of vitamin C on respiratory symptoms during and after heavy physical stress and, according to a meta-analysis, vitamin C administration decreased the incidence of respiratory symptoms in these studies by 52% (95% CI: 36% to 65%) [65, 66]. Three of the studies were conducted on marathon runners [67–69], one study used Canadian soldiers on a winter exercise [70], and one study was on schoolchildren in a skiing camp in the Swiss Alps [71]. In the general population, acute cough and sore throat usually indicates a viral etiology, and authors of these five trials assumed that the respiratory symptoms were caused by viruses. However, when such symptoms occur after a marathon run and other endurance sports sessions, they are not always caused by a viral infection. Instead they can result from an injury to the airway epithelium caused by hours of exceptional ventilatory exertion [1–3, 72]. Respiratory symptoms associated with physical irritation of the airways, allergy, and viral infections are all similar [66, 73], and therefore there is no justification to assume that all the respiratory symptoms in the above-mentioned five studies were caused by viruses. In one study, 70% of respiratory symptom episodes of elite athletes were caused by a non-infectious etiology, which indicates high prevalence of respiratory symptoms not caused by viruses in athletes [74].

In their study on marathon runners, Peters et al. [67] recorded the “self-reported symptoms including a running nose, sneezing, sore throat, cough” during a 2-week period after the race [67]. The incidence of post-race cough was reduced by 71% in the vitamin C group as compared to the placebo group (P[2-t] =0.014; 4/43 vs. 13/41). The incidence of sore throat was reduced by 67% in the vitamin C group (P =0.001; 8/43 vs. 23/41). In contrast, vitamin C had no significant effect on the incidence of runny nose (P =0.2; 13/43 vs. 19/41), which is the most bothersome symptom of rhinovirus infections [75]. Peters et al. did not carry out any virologic or pulmonary function tests and therefore the etiology of the cough and sore throat in the marathon runners is uncertain [67]. It is plausible that the common cold studies on marathon runners may have been partly measuring the effect of vitamin C on the epithelial injury caused by the heavy exertional ventilation.

A recent randomized trial in Israel found that vitamin C shortened the duration of respiratory symptoms in male adolescent competitive swimmers by 47% (95% CI: 14% to 80%), but no effect was seen in their female counterparts [76]. The difference found between the sexes in that study was significant (P =0.003). Here too, the etiology was not investigated and the respiratory symptoms might have been partly caused by the non-infectious irritation of swimmers’ airways [77].

Thus, in six randomized trials vitamin C reduced the incidence and duration of respiratory symptoms caused by heavy physical activity, yet it is not clear to what extent the symptoms were caused by non-infectious injury to the airway epithelium as opposed to viral infections. Irrespective of the etiology, symptoms are more important from the EBM point of view than laboratory measurements such as FEV_1_.

### Asthma phenotypes other than EIB

Asthma is a heterogeneous syndrome, an “umbrella concept,” that includes different phenotypes with different underlying pathophysiologies [78, 79]. Therefore, it is relevant to consider whether the effects of vitamin C are limited to EIB or whether vitamin C might also influence other asthma phenotypes. There is evidence that some virus infections cause a transient increase in oxidative stress [80, 81], and a systematic review identified three studies that indicated that vitamin C might protect against common cold-induced asthma [82].

A 4-month trial on 154 British asthmatics showed that the FEV_1_ level was not influenced by 1 g/day of vitamin C [83]. Moreover, the FEV_1_ level was not influenced by a 5-year administration of 0.25 g/day of vitamin C along with vitamin E and β-carotene in a large-scale trial on 20,536 British adults [84]. These two British-based studies imply that vitamin C supplementation does not influence pulmonary functions in patients with stable asthma or in relatively healthy people. Nevertheless, the study on British asthmatics found that the need for inhaled corticosteroids was lower in the vitamin C group [85]. In any case, vitamin C may beneficially influence pulmonary functions of some people under certain forms of acute stress, such as when they endure heavy physical activity or suffer from a viral respiratory tract infection.

### Refocusing research onto the biological effects of vitamin C

Vitamin C was identified in the search for the substance the deficiency of which led to scurvy. These early studies led to the assumption that the sole physiological function of vitamin C is just to prevent and treat scurvy. Therefore, it is often assumed that higher doses of vitamin C have no benefit when a person does not suffer from scurvy. In view of this strongly entrenched assumption, assessing the role of vitamin C on diseases and conditions other than scurvy is not just an empirical question but also a conceptual issue.

Bias against vitamin supplementation in general has been well documented [86–88]. Several influential reviews on vitamin C and the common cold have been shown to be erroneous and misleading [89, 90]. A Cochrane review on vitamin C and asthma [91] was shown to have substantial errors in the extraction of data and data analysis, and the review misled readers for a decade [92, 93]. If we wish for progress in the understanding of the effects of vitamin C on EIB, it needs to be acknowledged that the effects of vitamin C are not limited to the prevention of scurvy alone and, consequently, the published data on vitamin C should be analyzed carefully and comprehensively.

## Conclusions

Three trials have examined the effect of vitamin C on EIB and found that 0.5 to 2 g/day vitamin C halved the postexercise FEV_1_ decline (Figure 1). In addition, five trials found that vitamin C administration halved the incidence of respiratory symptoms after short-term heavy physical stress [65, 66] and one other trial found that vitamin C administration halved the duration of the respiratory symptoms in male adolescent competitive swimmers [76]. Although these nine randomized trials indicate that vitamin C has genuine biological effects on pulmonary functions and respiratory symptoms in people doing heavy exercise, it is unclear how the effect of vitamin C depends on the kind of physical activity, the level of physical activity, the temperature of ambient air, the humidity of ambient air, the dose of vitamin C, the level of dietary intake of vitamin C, and various other factors.

Given the safety and low cost of vitamin C, and the consistency of positive findings in the nine randomized trials on vitamin C against EIB and respiratory symptoms, it seems reasonable for physically active people to test whether vitamin C is beneficial on an individual basis, if they have documented EIB or suffer from respiratory symptoms such as cough or sore throat after taking vigorous exercise.

In future studies on vitamin C and EIB, a variety of pulmonary function outcomes should be measured. Although FEV_1_ is currently the standard measure for diagnosing EIB, the effect of vitamin C on the postexercise FEF_50_ and FEF_75_ levels should also be examined, as they may provide important additional information (Figures 2D and 3D). Furthermore, PEF is much easier to measure than carrying out a spirometry test. Therefore the possibility that the postexercise PEF decline might identify those people who may benefit from vitamin C administration should be investigated (Figures 2B and 3B).

In the earlier studies on vitamin C and EIB, only the mean effects on FEV_1_ were calculated [51, 56] or EIB was dichotomized by an arbitrary cut off level [57]. However, postexercise FEV_1_ decline is a continuous variable and the effect of vitamin C may depend on the baseline level of pulmonary function decline. Therefore, linear modelling is a much more informative way to analyze vitamin C effects on pulmonary functions and it should be used in the future studies (Figures 2 and 3). Finally, while pulmonary function tests are objective outcomes, symptoms are much more relevant outcomes from the EBM point of view, therefore both of them should be recorded concurrently in future investigations.

## Electronic supplementary material

Additional file 1: **Methods for Figure** 2 **(5 min data ) and Figures** 2 **E and** 2 **F.** (PDF 241 KB)

Additional file 2: **Methods for Figure** 3 **(0 min data) and Figures** 3 **E and** 3 **F.** (PDF 236 KB)

## Abbreviations

CI: Confidence interval
EBM: Evidence based medicine
EIB: Exercise-induced bronchoconstriction
FEF_25-75_: Mean forced expiratory flow between 25% and 75% of FVC
FEF_50_: Forced expiratory flow when 50% of the FVC has been expired
FEF_60_: Forced expiratory flow when 60% of the FVC has been expired
FEF_60(P)_: FEF_60_ value based on partial flow volume curves
FEF_75_: Forced expiratory flow when 75% of the FVC has been expired
FEV_1_: Forced expiratory volume in 1 second
FVC: Forced vital capacity
LT: Leukotriene
PEF: Peak expiratory flow
PG: Prostaglandin.
