# Supplementary material for: The effect of vitamin C on bronchoconstriction and respiratory symptoms caused by exercise: a review and statistical analysis
Source: Allergy Asthma Clin Immunol. 2014 Nov 27;10(1):58. doi: 10.1186/1710-1492-10-58 (PMC4363347; doi:10.1186/1710-1492-10-58)

Additional File 1

The effect of vitamin C on bronchoconstriction and respiratory symptoms caused by exercise: a review and statistical analysis

Harri Hemilä

Published in AACI
http://www.aacijournal.com/

This file describes the Methods for Fig. 2 (5 min) and shows Figs. 2E and 2F
Nov 18, 2014

Harri Hemilä
Department of Public Health
University of Helsinki
Helsinki, Finland
harri.hemila@helsinki.fi
http://www.mv.helsinki.fi/home/hemila/
http://www.mv.helsinki.fi/home/hemila/vitc_asthma.htm (papers on vitamin C and asthma)

This is a secondary analysis of the Schachter and Schlesinger (1982) study [S&S].
PubMed record of S&S:
http://www.ncbi.nlm.nih.gov/pubmed/7114587

For the purpose of checking,
S&S Table III (5 min after exercise) and Table V (baseline) are available at:
http://www.mv.helsinki.fi/home/hemila/A/Schachter.htm

Contents

Page

2 Definition of variables

3-7 R-program printouts for the analysis of the 5 min data
3 Fig. 2A: FEV_1 - 5-min data
4 Fig. 2B: PEF - 5-min data
5 Fig. 2C: FEF_{60} - 5-min data - effect on the postexercise FEF_{60} **change**
6 Fig. 2D: FEF_{60} - 5-min data - effect on the postexercise FEF_{60} **level**

8-10 Figures 2E and 2F
8 Fig. 2E: $FEF_{60(P)}$ - 5-min data - effect on the postexercise $FEF_{60(P)}$ **change**
8 Fig. 2F: $FEF_{60(P)}$ - 5-min data - effect on the postexercise $FEF_{60(P)}$ **level**
9-10 R-program printouts for Figs. 2E-F

11 Scatter plots of the pulmonary outcomes on vitamin C and placebo days – 5 min

DEFINITION OF VARIABLES

```
MEF40 is a term used by S&S. In the current terminology it corresponds to FEF60
MEF40(P) is named below as FEF60(P) [which means MEF40 measured from on partial flow volume curve]
```

Variables published by S&S:

```
NO      = Participant number in the S&S report
PlCh    = Placebo day:   Change in FEV1/PEF/FEF60/FEF60(P) 5 min after exercise (from S&S Table III)
VcCh    = Vitamin C day: Change in FEV1/PEF/FEF60/FEF60(P) 5 min after exercise (from S&S Table III)
PlBase  = Placebo day:   baseline (pre-exercise) FEV1/PEF/FEF60/FEF60(P) level  (from S&S Table V)
VcBase  = Vitamin C day: baseline (pre-exercise) FEV1/PEF/FEF60/FEF60(P) level  (from S&S Table V)
```

The following are calculated from the above data:

```
P_D         = change on the placebo day:    PlCh/PlBase - 1 (in % units)
VC_D        = change on the vitamin C day:  VcCh/VcBase - 1 (in % units)
Eff_VitC_x = Effect of vitamin C in percentage points: P_D - VC_D
              (x indicates FEV1/PEF/FEF60/FEF60(P))
Base_D      = Difference in baseline levels between placebo and vitamin C days: PlBase - VcBase
PlEnd       = Placebo day:   Postexercise level of FEF60/FEF60(P):  PlBase - PlCh
VcEnd       = Vitamin C day: Postexercise level of FEF60/FEF60(P):  VcBase - VcCh
End_Ratio   = Effect of vitamin C on postexercise FEF60/FEF60(P) level:
              (VcEnd/PlEnd) - 1 (in % units)
_ed         = Small changes are made in the data points to avoid overlapping in Figs
              (but the statistical models are based on the actual data and not on the edited data)
```

```
**************************************************************************************
```

The statistical models on the following pages were constructed by using the R-package:
http://www.r-project.org/

Likelihood ratio (LR) test was used to compare two statistical models to find out whether the addition of a new variable improves the model significantly.
For a short description of the LR test:
http://en.wikipedia.org/wiki/Likelihood-ratio_test
In the comparison of two statistical models, a large χ^2 value (a small P-value) means that the added variable significantly improves the regression model.

```
> # START Fig 2A: FEV1 -  5 minutes after exercise
> SchachterFEV1
   NO PlCh PlBase    P_D VcCh VcBase   VC_D Base_D VC_Eff_FEV1
1   1 -0.3    2.8 -10.71 -0.2    2.8  -7.14    0.0        3.57
2   2 -0.7    2.8 -25.00 -0.4    3.0 -13.33    0.2       11.67
3   3 -0.8    2.2 -36.36 -0.4    2.0 -20.00   -0.2       16.36
4   4 -0.9    2.4 -37.50 -0.1    2.1  -4.76   -0.3       32.74
5   5  0.0    2.9   0.00  0.0    2.4   0.00   -0.5        0.00
6   6  0.0    2.8   0.00 -0.3    2.7 -11.11   -0.1      -11.11
7   7  0.0    2.9   0.00 -0.1    2.3  -4.35   -0.6       -4.35
8   8 -0.1    2.1  -4.76  0.0    1.8   0.00   -0.3        4.76
9   9 -0.4    2.7 -14.81 -0.2    2.5  -8.00   -0.2        6.81
10 10  0.1    4.2   2.38  0.0    4.4   0.00    0.2       -2.38
11 11 -1.4    2.7 -51.85 -0.7    2.1 -33.33   -0.6       18.52
12 12 -0.8    2.5 -32.00 -0.5    2.5 -20.00    0.0       12.00

> # ABSOLUTE differences in FEV1 CHANGE at 5 min after exercise (Placebo - vit C), S&S Table III
> t.test(SchachterFEV1$PlCh, SchachterFEV1$VcCh, paired=TRUE, alternative='two.sided', mu=0.0,
conf.level=.95)
    Paired t-test
data:  SchachterFEV1$PlCh and SchachterFEV1$VcCh
t = -2.1335, df = 11, p-value = 0.05624
> # S&S reported t = 2.13 in Table III

> # ABSOLUTE difference in FEV1 BASELINE levels (Placebo - vit C = Base_D), S&S Table V
> t.test(SchachterFEV1$PlBase, SchachterFEV1$VcBase, paired=TRUE, alternative='two.sided', mu=0.0,
conf.level=.95)
    Paired t-test
data:  SchachterFEV1$PlBase and SchachterFEV1$VcBase
t = 2.5071, df = 11, p-value = 0.02913
> # S&S reported t = 2.51 in Table V

> # Modification of the vitamin C effect by the placebo-day postexercise FEV1 change (P_D)
> LinearModel.11 <- lm(VC_Eff_FEV1 ~ 1 ,      data=SchachterFEV1)
> summary(LinearModel.11)
Coefficients:
            Estimate Std. Error t value Pr(>|t|)
(Intercept)    7.383      3.414   2.162   0.0535 .
Residual standard error: 11.83 on 11 degrees of freedom

> LinearModel.12 <- lm(VC_Eff_FEV1 ~ 1 + P_D, data=SchachterFEV1)
> summary(LinearModel.12)
Coefficients:
            Estimate Std. Error t value Pr(>|t|)
(Intercept)  -2.3587     2.5400  -0.929 0.374966
P_D          -0.5550     0.1021  -5.437 0.000286 ***
Residual standard error: 6.237 on 10 degrees of freedom
Multiple R-squared: 0.7472, Adjusted R-squared: 0.7219
F-statistic: 29.56 on 1 and 10 DF,  p-value: 0.0002862

> confint(LinearModel.12)
                 2.5 %     97.5 %
(Intercept) -8.0182460  3.3008733
P_D         -0.7825026 -0.3275514

> # MODEL "12" ABOVE to Fig 2A

> # This LR-test below shows that there is significant improvement in the model fit
> # when the placebo-day postexercise FEV1 CHANGE is added
> lrtest(LinearModel.11,LinearModel.12)
Likelihood ratio test
Model 1: VC_Eff_FEV1 ~ 1
Model 2: VC_Eff_FEV1 ~ 1 + P_D
  #Df  LogLik Df  Chisq Pr(>Chisq)
1   2 -46.149
2   3 -37.899  1 16.502  4.861e-05 ***

> # There is a significant difference in the pre-exercise FEV1 levels on the two days (t = 2.51),
see above
> # This model below tests whether the inclusion of Base_D influences the estimate for P_D
> LinearModel.14 <- lm(VC_Eff_FEV1 ~ 1 + P_D + Base_D, data=SchachterFEV1)
> summary(LinearModel.14)
Coefficients:
            Estimate Std. Error t value Pr(>|t|)
(Intercept)  -2.2796     2.9318  -0.778 0.456784
P_D          -0.5560     0.1085  -5.123 0.000626 ***
Base_D        0.4784     7.2341   0.066 0.948719
Residual standard error: 6.572 on 9 degrees of freedom
Multiple R-squared: 0.7473, Adjusted R-squared: 0.6912
F-statistic: 13.31 on 2 and 9 DF,  p-value: 0.002049

> # The estimate for P_D is NOT substantially changed
```

```
> # START Fig 2B: PEF -  5 minutes after exercise

> SchachterPEF
   NO PlCh PlBase    P_D P_D_ed VcCh VcBase   VC_D VC_Eff_PEF VC_Eff_PEF_ed
1   1 -0.3    6.3  -4.76  -4.76 -0.3    6.1  -4.92      -0.16          0.34
2   2 -1.6    5.5 -29.09 -29.09 -0.9    5.7 -15.79      13.30         13.30
3   3 -1.1    4.9 -22.45 -22.45 -1.6    4.3 -37.21     -14.76        -14.76
4   4 -2.2    5.2 -42.31 -42.31  0.2    4.1   4.88      47.19         47.19
5   5  0.2    6.3   3.17   3.17 -0.8    6.3 -12.70     -15.87        -15.87
6   6 -0.2    5.9  -3.39  -2.99 -0.2    5.2  -3.85      -0.46         -0.46
7   7 -0.3    6.0  -5.00  -5.50 -0.3    5.3  -5.66      -0.66         -0.66
8   8  0.1    4.3   2.33   2.83  0.0    3.9   0.00      -2.33         -2.83
9   9 -0.7    6.1 -11.48 -11.48  0.0    5.4   0.00      11.48         11.48
10 10  0.0    9.1   0.00   0.00 -0.2    9.7  -2.06      -2.06         -1.56
11 11 -3.6    6.5 -55.38 -55.38 -3.2    5.1 -62.75      -7.36         -7.36
12 12 -1.7    5.7 -29.82 -29.82 -1.5    5.6 -26.79       3.04          3.04

> # ABSOLUTE difference in PEF CHANGES at 5 min after exercise (Placebo - vit C), S&S Table III
> t.test(SchachterPEF$PlCh, SchachterPEF$VcCh, paired=TRUE,  alternative='two.sided', mu=0.0, 
conf.level=.95)
    Paired t-test
data:  SchachterPEF$PlCh and SchachterPEF$VcCh
t = -0.8984, df = 11, p-value = 0.3882
> # S&S reported t = 0.90 in Table III

> # ABSOLUTE difference in PEF BASELINE levels (Placebo - vit C = Base_D), S&S Table V
> t.test(SchachterPEF$PlBase, SchachterPEF$VcBase, paired=TRUE,  alternative='two.sided', mu=0.0, 
conf.level=.95)
    Paired t-test
data:  SchachterPEF$PlBase and SchachterPEF$VcBase
t = 2.6317, df = 11, p-value = 0.02334
> # S&S reported t = 2.63 in Table V


> # Modification of the vitamin C effect by placebo-day postexercise PEF change (P_D)

> LinearModel.21 <- lm(VC_Eff_PEF ~ 1 ,      data=SchachterPEF)
> summary(LinearModel.21)
Coefficients:
            Estimate Std. Error t value Pr(>|t|)
(Intercept)    2.613      4.765   0.548    0.594
Residual standard error: 16.51 on 11 degrees of freedom


> LinearModel.22 <- lm(VC_Eff_PEF ~ 1 + P_D, data=SchachterPEF)
> summary(LinearModel.22)
Coefficients:
            Estimate Std. Error t value Pr(>|t|)
(Intercept)  -3.1221     6.1581  -0.507    0.623
P_D          -0.3472     0.2496  -1.391    0.194
Residual standard error: 15.85 on 10 degrees of freedom
Multiple R-squared: 0.1621,	Adjusted R-squared: 0.07834 
F-statistic: 1.935 on 1 and 10 DF,  p-value: 0.1944

> confint(LinearModel.22)
                  2.5 %    97.5 %
(Intercept) -16.8432615 10.599111
P_D          -0.9034366  0.208968

> # slope is NOT significantly different from the null effect and thus the model is not shown in 
Fig. 2B



> # This LR-test below shows that there is NOT significant improvement in the model fit
> # when the placebo-day postexercise PEF CHANGE is added
> lrtest(LinearModel.21,LinearModel.22)
Likelihood ratio test
Model 1: VC_Eff_PEF ~ 1
Model 2: VC_Eff_PEF ~ 1 + P_D
  #Df  LogLik Df  Chisq Pr(>Chisq)
1   2 -50.150                     
2   3 -49.089  1 2.1226     0.1451
```

> # START Fig 2C: FEF60 - 5 minutes after exercise RELATIVE CHANGE

> SchachterFEF60

	NO	PlCh	PlBase	P_D	VcCh	VcBase	VC_D	Base_D	VC_Eff_FEF60	PlEnd	VcEnd	End_Ratio	End_Ratio_**ed**	w09	w10	w11	w12	w13
1	1	-0.7	2.5	-28.00	-0.5	2.2	-22.73	-0.3	5.27	1.8	1.7	-5.56	-5.56	0.9	0.8	0.7	0.6	0.5
2	2	-0.9	1.4	-64.29	-0.4	1.4	-28.57	0.0	35.71	0.5	1.0	100.00	**103.00**	0.0	0.0	0.0	0.0	0.0
3	3	-0.7	1.1	-63.64	-0.3	0.9	-33.33	-0.2	30.30	0.4	0.6	50.00	50.00	0.0	0.0	0.0	0.0	0.0
4	4	-1.0	1.6	-62.50	0.0	1.2	0.00	-0.4	62.50	0.6	1.2	100.00	**97.00**	0.0	0.0	0.0	0.0	0.0
5	5	-0.2	1.8	-11.11	0.0	1.3	0.00	-0.5	11.11	1.6	1.3	-18.75	-18.75	0.7	0.6	0.5	0.4	0.3
6	6	0.0	2.8	0.00	-0.8	2.3	-34.78	-0.5	-34.78	2.8	1.5	-46.43	-46.43	1.9	1.8	1.7	1.6	1.5
7	7	-0.2	3.7	-5.41	-0.6	2.3	-26.09	-1.4	-20.68	3.5	1.7	-51.43	-51.43	2.6	2.5	2.4	2.3	2.2
8	8	0.0	1.1	0.00	-0.1	1.0	-10.00	-0.1	-10.00	1.1	0.9	-18.18	-18.18	0.2	0.1	0.0	0.0	0.0
9	9	-0.6	2.1	-28.57	-0.2	1.8	-11.11	-0.3	17.46	1.5	1.6	6.67	6.67	0.6	0.5	0.4	0.3	0.2
10	10	0.3	2.8	10.71	0.3	2.9	10.34	0.1	-0.37	3.1	3.2	3.23	3.23	2.2	2.1	2.0	1.9	1.8
11	11	-2.6	2.8	-92.86	-1.1	1.6	-68.75	-1.2	24.11	0.2	0.5	150.00	150.00	0.0	0.0	0.0	0.0	0.0
12	12	-1.2	1.6	-75.00	-0.9	1.8	-50.00	0.2	25.00	0.4	0.9	125.00	125.00	0.0	0.0	0.0	0.0	0.0

> # ABSOLUTE differences in FEF60 CHANGES at 5 min after exercise (Placebo - vit C), S&S Table III

```
> t.test(SchachterFEF60$PlCh, SchachterFEF60$VcCh, paired=TRUE, alternative='two.sided', mu=0.0,
conf.level=.95)
    Paired t-test
data:  SchachterFEF60$PlCh and SchachterFEF60$VcCh
t = -1.5422, df = 11, p-value = 0.1513
```

> # S&S reported t = 1.54 in Table III

> # ABSOLUTE difference in FEF60 BASELINE levels (Placebo - vit C = Base_D), S&S Table V

```
> t.test(SchachterFEF60$PlBase, SchachterFEF60$VcBase, paired=TRUE,  alternative='two.sided',
mu=0.0, conf.level=.95)
    Paired t-test
data:  SchachterFEF60$PlBase and SchachterFEF60$VcBase
t = 2.7437, df = 11, p-value = 0.01911
```

> # S&S reported t = 2.74 in Table V

> # Modification of the vitamin C effect by placebo-day postexercise FEF60 CHANGE (P_D)

```
> LinearModel.31 <- lm(VC_Eff_FEF60 ~ 1 ,      data=SchachterFEF60)
> summary(LinearModel.21)
Coefficients:
            Estimate Std. Error t value Pr(>|t|)
(Intercept)    2.613      4.765   0.548    0.594
Residual standard error: 16.51 on 11 degrees of freedom
```

```
> LinearModel.32 <- lm(VC_Eff_FEF60 ~ 1 + P_D, data=SchachterFEF60)
> summary(LinearModel.32)
Coefficients:
            Estimate Std. Error t value Pr(>|t|)
(Intercept)  -8.1840     7.4877  -1.093  0.30003
P_D          -0.5796     0.1544  -3.753  0.00377 **
Residual standard error: 17.92 on 10 degrees of freedom
Multiple R-squared: 0.5848, Adjusted R-squared: 0.5433
F-statistic: 14.09 on 1 and 10 DF,  p-value: 0.003765
```

> confint(LinearModel.32)

```
                  2.5 %     97.5 %
(Intercept) -24.8676798  8.4997601
P_D          -0.9237684 -0.2355133
```

> # MODEL "32" ABOVE is shown in Fig 2C

> # This LR-test below shows that there is significant improvement in the model fit
> # when the placebo-day postexercise FEF60 CHANGE is added

```
> lrtest(LinearModel.31,LinearModel.32)
Likelihood ratio test
Model 1: VC_Eff_FEF60 ~ 1
Model 2: VC_Eff_FEF60 ~ 1 + P_D
  #Df  LogLik Df  Chisq Pr(>Chisq)
1   2 -55.837
2   3 -50.562  1 10.548   0.001163 **
```

> # There is a significant difference in the pre-exercise FEF60 levels on the two days (t = 2.74), see above
> # This model below tests whether the inclusion of Base_D influences the estimate for P_D

```
> LinearModel.34 <- lm(VC_Eff_FEF60 ~ 1 + P_D + Base_D, data=SchachterFEF60)
> summary(LinearModel.34)
Coefficients:
            Estimate Std. Error t value Pr(>|t|)
(Intercept)  -2.6664     8.1977  -0.325  0.75242
P_D          -0.5841     0.1478  -3.951  0.00335 **
Base_D       14.8035    10.6841   1.386  0.19925
Residual standard error: 17.15 on 9 degrees of freedom
Multiple R-squared: 0.6578, Adjusted R-squared: 0.5818
F-statistic:  8.65 on 2 and 9 DF,  p-value: 0.008021
```

> # The estimate for P_D is NOT substantially changed

```
> # START Fig 2D: FEF60 - 5 minutes after exercise   ABSOLUTE LEVEL AFTER EXERCISE

> # Rationalization of the Spline modeling, see e.g.:
> # Greenland S. Dose-response and trend analysis in epidemiology: alternatives to categorical analysis.
> # Epidemiology. 1995 Jul;6(4):356-65.
> # http://www.ncbi.nlm.nih.gov/pubmed/7548341
> # http://dx.doi.org/10.1097/00001648-199507000-00005
> # and Wikipedia
> # http://en.wikipedia.org/wiki/Spline_(mathematics)

> # Modification of the vitamin C effect by the placebo-day postexercise FEF60 level (PlEnd)
> LinearModel.41 <- lm(End_Ratio ~ 1  , data=SchachterFEF60)
> summary(LinearModel.41)
Coefficients:
            Estimate Std. Error t value Pr(>|t|)
(Intercept)    32.88      20.08   1.637     0.13
Residual standard error: 69.56 on 11 degrees of freedom
> LinearModel.42 <- lm(End_Ratio ~ 1 + PlEnd  , data=SchachterFEF60)
> summary(LinearModel.42)
Coefficients:
            Estimate Std. Error t value Pr(>|t|)
(Intercept)   105.28      20.22   5.207 0.000397 ***
PlEnd         -49.65      11.08  -4.479 0.001181 **
Residual standard error: 42.08 on 10 degrees of freedom
Multiple R-squared: 0.6673, Adjusted R-squared: 0.6341
F-statistic: 20.06 on 1 and 10 DF,  p-value: 0.001181

> # This LR-test below shows that there is significant improvement in the model fit
> # when the placebo-day postexercise FEF60 level is added
> lrtest(LinearModel.41,LinearModel.42)
Likelihood ratio test
Model 1: End_Ratio ~ 1
Model 2: End_Ratio ~ 1 + PlEnd
  #Df  LogLik Df  Chisq Pr(>Chisq)
1   2 -67.411
2   3 -60.807  1 13.208  0.0002788 ***



> # These models below search for the optimal location of the spline knot
in the range 0.9 to 1.3 L/s

> SchachterFEF60$w09 <- with(SchachterFEF60, (PlEnd>0.9)*(PlEnd-0.9))
> LinearModel.409 <- lm(End_Ratio ~ 1 + PlEnd + w09 , data=SchachterFEF60)
> lrtest(LinearModel.42,LinearModel.409)
Likelihood ratio test
Model 1: End_Ratio ~ 1 + PlEnd
Model 2: End_Ratio ~ 1 + PlEnd + w09
  #Df  LogLik Df  Chisq Pr(>Chisq)
1   3 -60.807
2   4 -55.549  1 10.517   0.001183 **

> SchachterFEF60$w10 <- with(SchachterFEF60, (PlEnd>1.0)*(PlEnd-1.0))
> LinearModel.410 <- lm(End_Ratio ~ 1 + PlEnd + w10 , data=SchachterFEF60)
> lrtest(LinearModel.42,LinearModel.410)
Likelihood ratio test
Model 1: End_Ratio ~ 1 + PlEnd
Model 2: End_Ratio ~ 1 + PlEnd + w10
  #Df  LogLik Df  Chisq Pr(>Chisq)
1   3 -60.807
2   4 -55.201  1 11.212  0.0008126 ***
```

```
> SchachterFEF60$w11 <- with(SchachterFEF60, (PlEnd>1.1)*(PlEnd-1.1))
> LinearModel.411 <- lm(End_Ratio ~ 1 + PlEnd + w11 , data=SchachterFEF60)
> summary(LinearModel.411)
Coefficients:
            Estimate Std. Error t value Pr(>|t|)
(Intercept)   170.17      21.47   7.925 2.39e-05 ***
PlEnd        -157.53      29.12  -5.409 0.000428 ***
w11           144.05      37.68   3.824 0.004068 **
Residual standard error: 27.38 on 9 degrees of freedom
Multiple R-squared: 0.8732, Adjusted R-squared: 0.8451
F-statistic:    31 on 2 and 9 DF,  p-value: 9.191e-05
> lrtest(LinearModel.42,LinearModel.411)
Likelihood ratio test
Model 1: End_Ratio ~ 1 + PlEnd
Model 2: End_Ratio ~ 1 + PlEnd + w11
  #Df  LogLik Df  Chisq Pr(>Chisq)
1   3 -60.807
2   4 -55.018  1 11.578  0.0006673 ***


> # Spline Knot at 1.1 L/s gives the best model as indicated by the largest chi-square
> # MODEL "411" ABOVE is shown in Fig 2D


> # The LR-test below tests the improvement of the model fit
> # by the addition of the 2 spline segments to the null model containing only the intercept
> lrtest(LinearModel.41,LinearModel.411)
Likelihood ratio test
Model 1: End_Ratio ~ 1
Model 2: End_Ratio ~ 1 + PlEnd + w11
  #Df  LogLik Df  Chisq Pr(>Chisq)
1   2 -67.411
2   4 -55.018  2 24.786  4.148e-06 ***


> # In the model below, PlEnd is restricted to the 7 participants with FEF60 above 1 L/s
> # this group does not differ from null effect
> # using subset function
> Sch_Over11 <- subset(SchachterFEF60, PlEnd>=1.1)
> LinearModel.411b <- lm(End_Ratio ~ 1 + PlEnd , data=Sch_Over11 )
> summary(LinearModel.411b)
Coefficients:
            Estimate Std. Error t value Pr(>|t|)
(Intercept)    8.651     22.722   0.381    0.719
PlEnd        -12.403      9.631  -1.288    0.254
Residual standard error: 21.71 on 5 degrees of freedom
Multiple R-squared: 0.2491, Adjusted R-squared: 0.09887
F-statistic: 1.658 on 1 and 5 DF,  p-value: 0.2542
```

```
> SchachterFEF60$w12 <- with(SchachterFEF60, (PlEnd>1.2)*(PlEnd-1.2))
> LinearModel.412 <- lm(End_Ratio ~ 1 + PlEnd + w12 , data=SchachterFEF60)
> lrtest(LinearModel.42,LinearModel.412)
Likelihood ratio test
Model 1: End_Ratio ~ 1 + PlEnd
Model 2: End_Ratio ~ 1 + PlEnd + w12
  #Df  LogLik Df  Chisq Pr(>Chisq)
1   3 -60.807
2   4 -55.361  1 10.893  0.0009653 ***


> SchachterFEF60$w13 <- with(SchachterFEF60, (PlEnd>1.3)*(PlEnd-1.3))
> LinearModel.413 <- lm(End_Ratio ~ 1 + PlEnd + w13 , data=SchachterFEF60)
> lrtest(LinearModel.42,LinearModel.413)
Likelihood ratio test
Model 1: End_Ratio ~ 1 + PlEnd
Model 2: End_Ratio ~ 1 + PlEnd + w13
  #Df  LogLik Df  Chisq Pr(>Chisq)
1   3 -60.807
2   4 -55.796  1 10.022   0.001547 **
```

Figs. 2E-F. Effect of vitamin C on the 5-min postexercise $FEF_{60(P)}$

In Figs. 2E and 2F the dash lines indicate equality between vitamin C and placebo.
If vitamin C has no effect, the observations would be located randomly and symmetrically on both sides of the dash lines. The continuous lines indicate the regression lines.
The numbers identify the participants with the same numbers as used by S&S.

2E: Vitamin C effect on postexercise $FEF_{60(P)}$ change.
This figure shows the effect of vitamin C in percentage points (pp).
For example, on the placebo-day, participant #11 had a $FEF_{60(P)}$ change of -93.33%, and on the vitamin C day a $FEF_{60(P)}$ change of -66.67%, which gives the 27 pp improvement shown in Fig. 2E.
Slope of the linear regression model indicates that postexercise $FEF_{60(P)}$ change was decreased by 63% (95% CI: 29% to 97%) on the vitamin C day compared with the change on the placebo day.
Addition of the placebo-day postexercise $FEF_{60(P)}$ change to the model containing the intercept improved the model fit by χ^2(1 df) = 12.0 (P = 0.0005).

2F: Vitamin C effect on postexercise $FEF_{60(P)}$ level.
This figure shows the effect of vitamin C in percentages.
For example, on the placebo-day, participant #11 had a postexercise $FEF_{60(P)}$ level of 0.2 L/s, and on the vitamin C day a postexercise $FEF_{60(P)}$ level of 0.6 L/s, which gives the 200% increase in the postexercise $FEF_{60(P)}$ level shown in Fig. 2F.
Addition of the placebo-day postexercise $FEF_{60(P)}$ level to the model containing the intercept improved the model fit by χ^2(1 df) = 9.7 (P = 0.002). Addition of the spline knot at 1.1 L/s further improved the fit by χ^2(1 df) = 15.9 (P = 0.0001). Simultaneous addition of the two spline segments with the knot at 1.1 L/s to the model containing only the intercept improved the model fit by χ^2(2 df) = 25.6 (P = 0.000003).

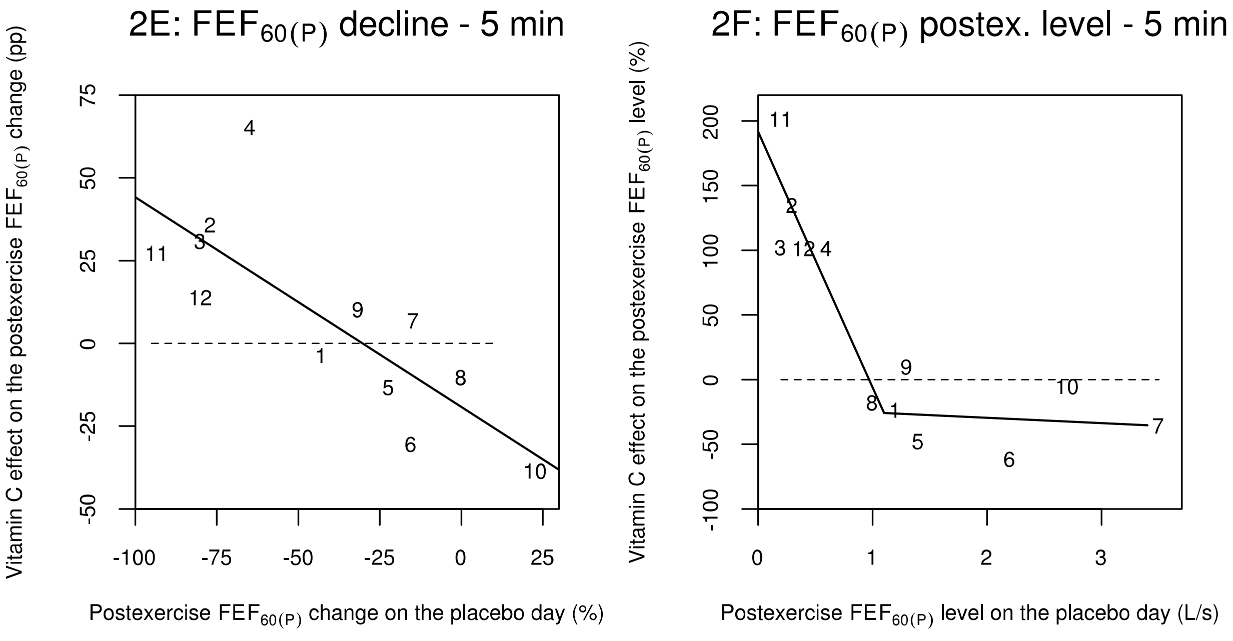

```
> # START Fig 2E : FEF60(P) 5 minutes after exercise    RELATIVE CHANGE
> SchachterFEF60_P
   NO PlCh PlBase    P_D VcCh VcBase   VC_D Base_D VC_Eff_FEF60P PlEnd VcEnd End_Ratio w10 w11 w12
1   1 -0.9    2.1 -42.86 -0.8    1.7 -47.06   -0.4         -4.20   1.2   0.9    -25.00 0.2 0.1 0.0
2   2 -1.0    1.3 -76.92 -0.5    1.2 -41.67   -0.1         35.26   0.3   0.7    133.33 0.0 0.0 0.0
3   3 -0.8    1.0 -80.00 -0.4    0.8 -50.00   -0.2         30.00   0.2   0.4    100.00 0.0 0.0 0.0
4   4 -1.1    1.7 -64.71  0.0    1.2   0.00   -0.5         64.71   0.6   1.2    100.00 0.0 0.0 0.0
5   5 -0.4    1.8 -22.22 -0.4    1.1 -36.36   -0.7        -14.14   1.4   0.7    -50.00 0.4 0.3 0.2
6   6 -0.4    2.6 -15.38 -0.7    1.5 -46.67   -1.1        -31.28   2.2   0.8    -63.64 1.2 1.1 1.0
7   7 -0.6    4.1 -14.63 -0.2    2.4  -8.33   -1.7          6.30   3.5   2.2    -37.14 2.5 2.4 2.3
8   8  0.0    1.0   0.00 -0.1    0.9 -11.11   -0.1        -11.11   1.0   0.8    -20.00 0.0 0.0 0.0
9   9 -0.6    1.9 -31.58 -0.4    1.8 -22.22   -0.1          9.36   1.3   1.4      7.69 0.3 0.2 0.1
10 10  0.5    2.2  22.73 -0.5    3.0 -16.67    0.8        -39.39   2.7   2.5     -7.41 1.7 1.6 1.5
11 11 -2.8    3.0 -93.33 -1.2    1.8 -66.67   -1.2         26.67   0.2   0.6    200.00 0.0 0.0 0.0
12 12 -1.6    2.0 -80.00 -1.6    2.4 -66.67    0.4         13.33   0.4   0.8    100.00 0.0 0.0 0.0

> # ABSOLUTE difference in FEF60(P) CHANGE at 5 min after exercise, S&S Table III
> t.test(SchachterFEF60_P$PlCh,   SchachterFEF60_P$VcCh,   paired=TRUE, alternative='two.sided',
mu=0.0, conf.level=.95)
    Paired t-test
data:  SchachterFEF60_P$PlCh and SchachterFEF60_P$VcCh
t = -1.2687, df = 11, p-value = 0.2307
> # S&S reported t = 1.27 in Table III

> # ABSOLUTE difference in FEF60(P) BASELINE levels (Base_D), S&S Table V
> t.test(SchachterFEF60_P$PlBase, SchachterFEF60_P$VcBase, paired=TRUE, alternative='two.sided',
mu=0.0, conf.level=.95)
    Paired t-test
data:  SchachterFEF60_P$PlBase and SchachterFEF60_P$VcBase
t = 2.0361, df = 11, p-value = 0.06655
> # S&S reported t = 2.04 in Table V

> # Modification of the vitamin C effect by the placebo-day postexercise FEF60(P) CHANGE (P_D)
> LinearModel.51 <- lm(VC_Eff_FEF60P ~ 1 ,      data=SchachterFEF60_P)
> summary(LinearModel.51)
Coefficients:
            Estimate Std. Error t value Pr(>|t|)
(Intercept)    7.126      8.532   0.835    0.421
Residual standard error: 29.56 on 11 degrees of freedom

> LinearModel.52 <- lm(VC_Eff_FEF60P ~ 1 + P_D, data=SchachterFEF60_P)
> summary(LinearModel.52)
Coefficients:
            Estimate Std. Error t value Pr(>|t|)
(Intercept) -19.2088     8.3367  -2.304  0.04394 *
P_D          -0.6334     0.1524  -4.156  0.00196 **
Residual standard error: 18.77 on 10 degrees of freedom
Multiple R-squared: 0.6334, Adjusted R-squared: 0.5967
F-statistic: 17.28 on 1 and 10 DF,  p-value: 0.00196

> confint(LinearModel.52)
                  2.5 %     97.5 %
(Intercept) -37.7841619 -0.6335135
P_D          -0.9729888 -0.2938625

> # MODEL "52" ABOVE is shown in Fig 2E in this Additional file 1

> # This LR-test below shows that there is significant improvement in the model fit
> # when the placebo-day postexercise FEF60(P) CHANGE is added
> lrtest(LinearModel.51,LinearModel.52)
Likelihood ratio test
Model 1: VC_Eff_FEF60P ~ 1
Model 2: VC_Eff_FEF60P ~ 1 + P_D
  #Df LogLik Df  Chisq Pr(>Chisq)
1   2 -57.14
2   3 -51.12  1 12.041  0.0005205 ***

> # There is a marginally nonsignificant difference in the pre-exercise FEF60P levels on the two
days (t = 2.04), see above
> # This model below tests whether the inclusion of Base_D changes the estimate for P_D
> LinearModel.54 <- lm(VC_Eff_FEF60P ~ 1 + P_D + Base_D, data=SchachterFEF60_P)
> summary(LinearModel.54)
Coefficients:
            Estimate Std. Error t value Pr(>|t|)
(Intercept) -19.9562     9.2054  -2.168  0.05831 .
P_D          -0.6292     0.1608  -3.913  0.00355 **
Base_D       -2.2568     8.5961  -0.263  0.79882
Residual standard error: 19.71 on 9 degrees of freedom
Multiple R-squared: 0.6362, Adjusted R-squared: 0.5553
F-statistic: 7.868 on 2 and 9 DF,  p-value: 0.01057

> # The estimate for P_D is NOT substantially changed
```

```
> # START Fig 2F : FEF60(P) 5 minutes after exercise  ABSOLUTE LEVEL AFTER EXERCISE

> # Modification of vitamin C effect by the placebo-day postexercise FEF60(P) level (PlEnd)
> LinearModel.61 <- lm(End_Ratio ~ 1  ,        data=SchachterFEF60_P)
> summary(LinearModel.61)
Coefficients:
            Estimate Std. Error t value Pr(>|t|)
(Intercept)    36.49      24.75   1.474    0.168
Residual standard error: 85.74 on 11 degrees of freedom
> LinearModel.62 <- lm(End_Ratio ~ 1 + PlEnd  , data=SchachterFEF60_P)
> summary(LinearModel.62)
Coefficients:
            Estimate Std. Error t value Pr(>|t|)
(Intercept)   111.76      27.40   4.079  0.00222 **
PlEnd         -60.22      17.00  -3.542  0.00534 **
Residual standard error: 59.89 on 10 degrees of freedom
Multiple R-squared: 0.5564, Adjusted R-squared: 0.5121
F-statistic: 12.55 on 1 and 10 DF,  p-value: 0.00534

> # This LR-test below shows that there is significant improvement in the model fit
> # when the placebo-day postexercise FEF60(P) level is added
> lrtest(LinearModel.61,LinearModel.62)
Likelihood ratio test
Model 1: End_Ratio ~ 1
Model 2: End_Ratio ~ 1 + PlEnd
  #Df  LogLik Df  Chisq Pr(>Chisq)
1   2 -69.921
2   3 -65.044  1 9.7552   0.001788 **


> # Model with the spline knot at 1.1 L/s

> SchachterFEF60_P$w11 <- with(SchachterFEF60_P, (PlEnd>1.1)*(PlEnd-1.1))
> LinearModel.611 <- lm(End_Ratio ~ 1 + PlEnd + w11 , data=SchachterFEF60_P)
> summary(LinearModel.611)
Coefficients:
            Estimate Std. Error t value Pr(>|t|)
(Intercept)   191.79      21.89   8.762 1.06e-05 ***
PlEnd        -197.90      29.11  -6.798 7.93e-05 ***
w11           193.83      38.87   4.987 0.000752 ***
Residual standard error: 32.54 on 9 degrees of freedom
Multiple R-squared: 0.8821, Adjusted R-squared: 0.8559
F-statistic: 33.68 on 2 and 9 DF,  p-value: 6.626e-05
> lrtest(LinearModel.62,LinearModel.611)
Likelihood ratio test
Model 1: End_Ratio ~ 1 + PlEnd
Model 2: End_Ratio ~ 1 + PlEnd + w11
  #Df  LogLik Df  Chisq Pr(>Chisq)
1   3 -65.044
2   4 -57.092  1 15.903  6.666e-05 ***


> # MODEL "611" ABOVE is shown in Fig 2F in this Additional file 1


> # The LR-test below tests the improvement of the model fit
> # by the addition of the 2 spline segments to the null model containing only the intercept
> lrtest(LinearModel.61,LinearModel.611)
Likelihood ratio test
Model 1: End_Ratio ~ 1
Model 2: End_Ratio ~ 1 + PlEnd + w11
  #Df  LogLik Df  Chisq Pr(>Chisq)
1   2 -69.921
2   4 -57.092  2 25.659  2.681e-06 ***
```

Scatter plots of the pulmonary outcomes on vitamin C and placebo days – 5 min

The following figures show the scatter plots for the same variables as Figs. 2A to 2F on the vitamin C and placebo days, in the same order.

The dash line indicates the identity between vitamin C and placebo. If vitamin C did not differ from the placebo, the data points would be located symmetrically on both sides of the dash line.

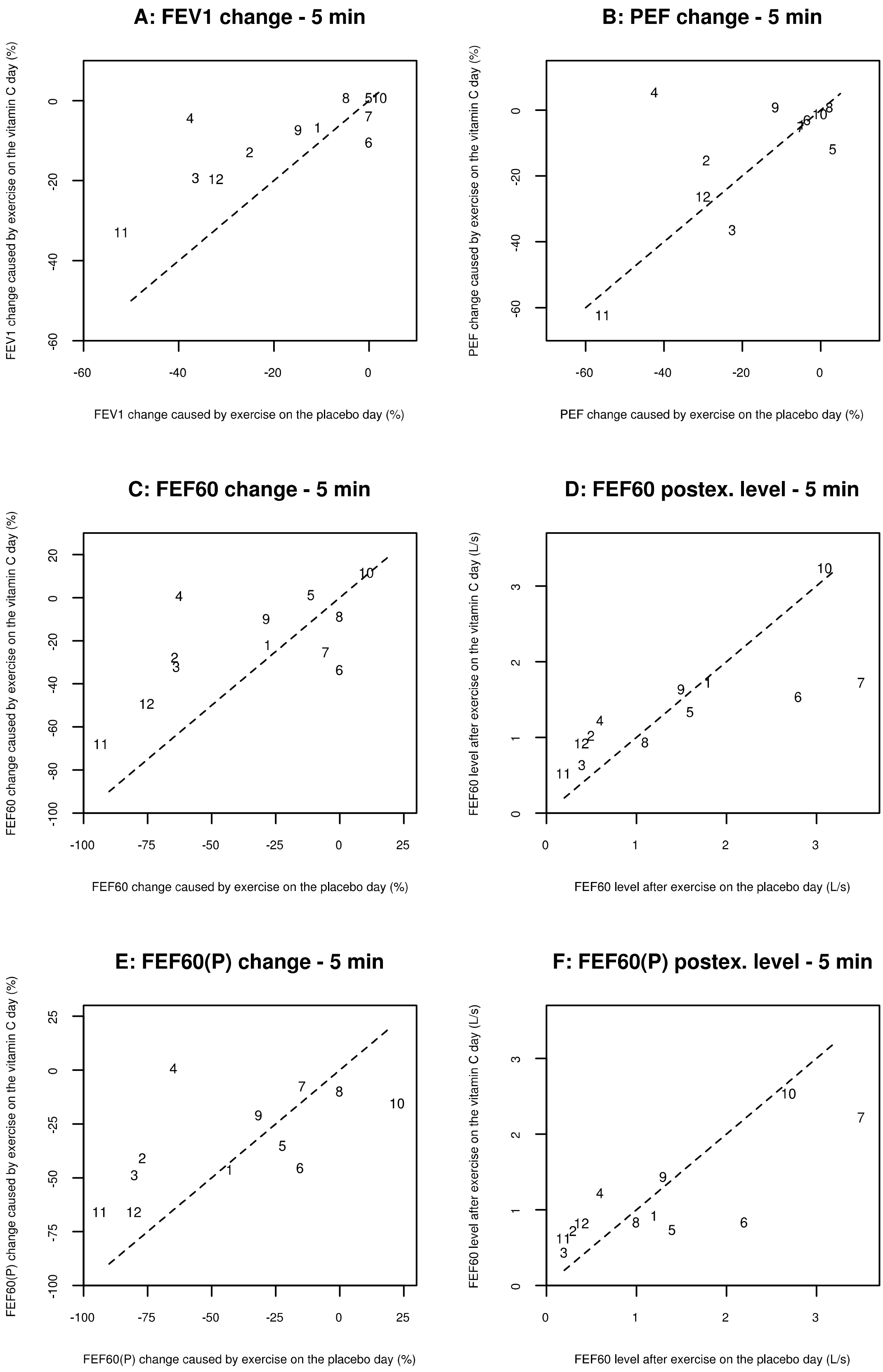

Supplement: Supplementary file 1 — Additional file 1: Methods for Figure 2 (5 min data ) and Figures 2 E and 2 F. (PDF 241 KB) [file 13223_2014_588_MOESM1_ESM.pdf]
