# Supplementary material for: The effect of vitamin C on bronchoconstriction and respiratory symptoms caused by exercise: a review and statistical analysis
Source: Allergy Asthma Clin Immunol. 2014 Nov 27;10(1):58. doi: 10.1186/1710-1492-10-58 (PMC4363347; doi:10.1186/1710-1492-10-58)

Additional File 2

The effect of vitamin C on bronchoconstriction and respiratory symptoms caused by exercise: a review and statistical analysis

Harri Hemilä

Published in AACI
http://www.aacijournal.com/

This file describes the Methods for Fig. 3 (0 min) and shows Figs. 3E and 3F
Nov 18, 2014

Harri Hemilä
Department of Public Health
University of Helsinki
Helsinki, Finland
harri.hemila@helsinki.fi
http://www.mv.helsinki.fi/home/hemila/
http://www.mv.helsinki.fi/home/hemila/vitc_asthma.htm (papers on vitamin C and asthma)

This is a secondary analysis of the Schachter and Schlesinger (1982) study [S&S].
PubMed record of S&S:
http://www.ncbi.nlm.nih.gov/pubmed/7114587

For the purpose of checking,
S&S Table II (0 min after exercise) and Table V (baseline) are available at:
http://www.mv.helsinki.fi/home/hemila/A/Schachter.htm

Contents

Page

2 Definition of variables

3-7 R-program printouts for the analysis of the 0 min data
3 Fig. 3A: FEV_1 - 0-min data
4 Fig. 3B: PEF - 0-min data
5 Fig. 3C: FEF_{60} - 0-min data - effect on the postexercise FEF_{60} **change**
6 Fig. 3D: FEF_{60} - 0-min data - effect on the postexercise FEF_{60} **level**

7-9 Figures 3E and 3F
7 Fig. 3C: $FEF_{60(P)}$ - 0-min data - effect on the postexercise $FEF_{60(P)}$ **change**
7 Fig. 3D: $FEF_{60(P)}$ - 0-min data - effect on the postexercise $FEF_{60(P)}$ **level**
8-9 R-program printouts for Figs. 3E-F

10 Scatter plots of the pulmonary outcomes on vitamin C and placebo days – 0 min

DEFINITION OF VARIABLES

```
MEF40 is a term used by S&S. In the current terminology it corresponds to FEF60
MEF40(P) is named below as FEF60(P), which means MEF40 based on a partial flow volume curve
```

Variables published by S&S:

```
NO      = Participant number in the S&S report
PlCh    = Placebo day:   Change in FEV1/PEF/FEF60/FEF60(P) 0 min after exercise (from S&S Table II)
VcCh    = Vitamin C day: Change in FEV1/PEF/FEF60/FEF60(P) 0 min after exercise (from S&S Table II)
PlBase  = Placebo day:   baseline (pre-exercise) FEV1/PEF/FEF60/FEF60(P) level  (from S&S Table V)
VcBase  = Vitamin C day: baseline (pre-exercise) FEV1/PEF/FEF60/FEF60(P) level  (from S&S Table V)
```

The following are calculated from the above data:

```
P_D         = change on the placebo day:    PlCh/PlBase - 1 (in % units)
VC_D        = change on the vitamin C day:  VcCh/VcBase - 1 (in % units)
Eff_VitC_x = Effect of vitamin C in percentage points: P_D - VC_D
              (x indicates FEV1/PEF/FEF60/FEF60(P))
Base_D      = Difference in baseline levels between placebo and vitamin C days: PlBase - VcBase
PlEnd       = Placebo day:   Postexercise level of FEF60/FEF60(P):  PlBase - PlCh
VcEnd       = Vitamin C day: Postexercise level of FEF60/FEF60(P):  VcBase - VcCh
End_Ratio   = Effect of vitamin C on postexercise FEF60/FEF60(P) level:
              (VcEnd/PlEnd) - 1 (in % units)
_ed         = Small changes are made in the data points to avoid overlapping in Figs
              (but the statistical models are based on the actual data and not on the edited data)
```

****

The statistical models on the following pages were constructed by using the R-package: http://www.r-project.org/

Likelihood ratio (LR) test was used to compare two statistical models to find out whether the addition of a new variable improves the model significantly.
For a short description of the LR test:
http://en.wikipedia.org/wiki/Likelihood-ratio_test
In the comparison of two statistical models, a large χ^2 value (a small P-value) means that the added variable significantly improves the regression model.

```
> # START Fig 3A : FEV1  -  0 minutes after exercise
> SchachterFEV1_0
   NO PlCh PlBase    P_D VcCh VcBase  VC_D Base_D VC_Eff_FEV1
1   1  0.1    2.8   3.57 -0.1    2.8 -3.57    0.0       -7.14
2   2  0.0    2.8   0.00  0.2    3.0  6.67    0.2        6.67
3   3 -0.1    2.2  -4.55  0.2    2.0 10.00   -0.2       14.55
4   4 -0.3    2.4 -12.50  0.2    2.1  9.52   -0.3       22.02
5   5  0.6    2.9  20.69  0.4    2.4 16.67   -0.5       -4.02
6   6  0.2    2.8   7.14  0.0    2.7  0.00   -0.1       -7.14
7   7  0.2    2.9   6.90  0.3    2.3 13.04   -0.6        6.15
8   8  0.1    2.1   4.76  0.4    1.8 22.22   -0.3       17.46
9   9  0.3    2.7  11.11  0.4    2.5 16.00   -0.2        4.89
10 10  0.4    4.2   9.52  0.2    4.4  4.55    0.2       -4.98
11 11 -0.3    2.7 -11.11  0.4    2.1 19.05   -0.6       30.16
12 12 -0.2    2.5  -8.00 -0.1    2.5 -4.00    0.0        4.00

> # absolute differences in FEV1 CHANGE at 0 min after exercise (Placebo - vit C), S&S Table II
> t.test(SchachterFEV1_0$PlCh, SchachterFEV1_0$VcCh, paired=TRUE, alternative='two.sided', mu=0.0,
conf.level=.95)
    Paired t-test
data:  SchachterFEV1_0$PlCh and SchachterFEV1_0$VcCh
t = -1.4639, df = 11, p-value = 0.1712
> # S&S reported t = 1.46 in Table II

> # absolute difference in FEV1 BASELINE levels (Placebo - vit C = Base_D), S&S Table V
> t.test(SchachterFEV1_0$PlBase, SchachterFEV1_0$VcBase, paired=TRUE, alternative='two.sided',
mu=0.0, conf.level=.95)
    Paired t-test
data:  SchachterFEV1_0$PlBase and SchachterFEV1_0$VcBase
t = 2.5071, df = 11, p-value = 0.02913
> # S&S reported t = 2.51 in Table V

> # Modification of the vitamin C effect by the placebo-day postexercise FEV1 change (P_D)

> LinearModel.11 <- lm(VC_Eff_FEV1 ~ 1       , data=SchachterFEV1_0)
> summary(LinearModel.11)
Coefficients:
            Estimate Std. Error t value Pr(>|t|)
(Intercept)    6.885      3.490   1.973   0.0741 .

> LinearModel.12 <- lm(VC_Eff_FEV1 ~ 1 + P_D , data=SchachterFEV1_0)
> summary(LinearModel.12)
Coefficients:
            Estimate Std. Error t value Pr(>|t|)
(Intercept)   8.8553     2.6777   3.307  0.00792 **
P_D          -0.8588     0.2747  -3.127  0.01075 *
Residual standard error: 9.015 on 10 degrees of freedom
Multiple R-squared: 0.4944, Adjusted R-squared: 0.4438
F-statistic: 9.777 on 1 and 10 DF,  p-value: 0.01075

> confint(LinearModel.12)
                2.5 %     97.5 %
(Intercept)  2.889072 14.8214751
P_D         -1.470800 -0.2468378

> # MODEL "12" ABOVE is shown in Fig 3A

> # This LR-test below shows that there is significant improvement in the model fit
> # when the placebo-day postexercise FEV1 change is added
> lrtest(LinearModel.11,LinearModel.12)
Likelihood ratio test
Model 1: VC_Eff_FEV1 ~ 1
Model 2: VC_Eff_FEV1 ~ 1 + P_D
  #Df  LogLik Df  Chisq Pr(>Chisq)
1   2 -46.412
2   3 -42.320  1 8.1833   0.004228 **
> # There is a significant difference in the pre-exercise FEV1 levels on the two days (t = 2.51),
see above
> # This model below tests whether the inclusion of Base_D influences the estimate for P_D

> LinearModel.13 <- lm(VC_Eff_FEV1 ~ 1 + P_D + Base_D, data=SchachterFEV1_0)
> summary(LinearModel.13)
Coefficients:
            Estimate Std. Error t value Pr(>|t|)
(Intercept)   4.5677     2.5339   1.803  0.10495
P_D          -0.8611     0.2096  -4.109  0.00264 **
Base_D      -21.4635     7.5047  -2.860  0.01878 *
Residual standard error: 6.878 on 9 degrees of freedom
Multiple R-squared: 0.7351, Adjusted R-squared: 0.6762
F-statistic: 12.49 on 2 and 9 DF,  p-value: 0.002534

> # The estimate for P_D is NOT substantially changed
```

```
> # START Fig 3B: PEF  -  0 minutes after exercise
> SchachterPEF_0
   NO PlCh PlBase    P_D VcCh VcBase  VC_D Base_D VC_Eff_PEF
1   1  0.9    6.3  14.29  0.4    6.1  6.56   -0.2      -7.73
2   2 -0.2    5.5  -3.64  0.0    5.7  0.00    0.2       3.64
3   3 -0.2    4.9  -4.08  0.7    4.3 16.28   -0.6      20.36
4   4 -1.0    5.2 -19.23  0.4    4.1  9.76   -1.1      28.99
5   5  1.6    6.3  25.40  1.4    6.3 22.22    0.0      -3.17
6   6  0.3    5.9   5.08  0.2    5.2  3.85   -0.7      -1.24
7   7  0.3    6.0   5.00  0.3    5.3  5.66   -0.7       0.66
8   8  0.4    4.3   9.30  0.9    3.9 23.08   -0.4      13.77
9   9  0.5    6.1   8.20  1.0    5.4 18.52   -0.7      10.32
10 10  0.8    9.1   8.79  1.3    9.7 13.40    0.6       4.61
11 11 -1.3    6.5 -20.00  0.9    5.1 17.65   -1.4      37.65
12 12 -0.9    5.7 -15.79 -0.4    5.6 -7.14   -0.1       8.65

> # absolute difference in PEF CHANGES at 0 min after exercise (Placebo - vit C), S&S Table II
> t.test(SchachterPEF_0$PlCh, SchachterPEF_0$VcCh, paired=TRUE,  alternative='two.sided', mu=0.0,
conf.level=.95)
    Paired t-test
data:  SchachterPEF_0$PlCh and SchachterPEF_0$VcCh
t = -2.2967, df = 11, p-value = 0.04227
> # S&S reported t = 2.3 in Table II

> # absolute difference in PEF BASELINE levels (Placebo - vit C =  = Base_D), S&S Table V
> t.test(SchachterPEF_0$PlBase, SchachterPEF_0$VcBase, paired=TRUE,  alternative='two.sided',
mu=0.0, conf.level=.95)
    Paired t-test
data:  SchachterPEF_0$PlBase and SchachterPEF_0$VcBase
t = 2.6317, df = 11, p-value = 0.02334
> # S&S reported t = 2.63 in Table V

> # Modification of the vitamin C effect by the placebo-day postexercise PEF change (P_D)

> LinearModel.21 <- lm(VC_Eff_PEF ~ 1       , data=SchachterPEF_0)
> summary(LinearModel.21)
Coefficients:
            Estimate Std. Error t value Pr(>|t|)
(Intercept)    9.709      3.910   2.483   0.0304 *

> LinearModel.22 <- lm(VC_Eff_PEF ~ 1 + P_D , data=SchachterPEF_0)
> summary(LinearModel.22)
Coefficients:
            Estimate Std. Error t value Pr(>|t|)
(Intercept)  10.5363     2.6169   4.026  0.00241 **
P_D          -0.7451     0.1942  -3.838  0.00328 **
Residual standard error: 9.035 on 10 degrees of freedom
Multiple R-squared: 0.5956, Adjusted R-squared: 0.5552
F-statistic: 14.73 on 1 and 10 DF,  p-value: 0.003277

> confint(LinearModel.22)
                2.5 %     97.5 %
(Intercept)  4.705327 16.3671737
P_D         -1.177716 -0.3125247

> # MODEL "22" ABOVE is shown in Fig 3B

> # This LR-test below shows that there is significant improvement in the model fit
> # when the placebo-day postexercise PEF change is added
> lrtest(LinearModel.21,LinearModel.22)
Likelihood ratio test
Model 1: VC_Eff_PEF ~ 1
Model 2: VC_Eff_PEF ~ 1 + P_D
  #Df  LogLik Df  Chisq Pr(>Chisq)
1   2 -47.778
2   3 -42.346  1 10.865  0.0009801 ***

> # There is a significant difference in the pre-exercise PEF levels on the two days (t = 2.63),
see above
> # This model below tests whether the inclusion of Base_D influences the estimate for P_D
> LinearModel.23 <- lm(VC_Eff_PEF ~ 1 + P_D + Base_D, data=SchachterPEF_0)
> summary(LinearModel.23)
Coefficients:
            Estimate Std. Error t value Pr(>|t|)
(Intercept)   6.5568     3.3320   1.968   0.0806 .
P_D          -0.5644     0.2065  -2.734   0.0231 *
Base_D       -8.8913     5.1779  -1.717   0.1201
Residual standard error: 8.265 on 9 degrees of freedom
Multiple R-squared: 0.6954, Adjusted R-squared: 0.6277
F-statistic: 10.27 on 2 and 9 DF,  p-value: 0.00475

> # The estimate for P_D is NOT substantially changed (0.74 vs. 0.56 is not great in this context)
```

```
> # START Fig 3C: FEF60  -  0 minutes after exercise   RELATIVE CHANGE
> SchachterFEF60_0
   NO PlCh PlBase    P_D VcCh VcBase   VC_D Base_D VC_Eff_FEF60 PlEnd VcEnd VcEnd_ed End_Ratio
1   1  0.4    2.5  16.00 -0.1    2.2  -4.55   -0.3       -20.55   2.9   2.1     2.10    -27.59
2   2  0.0    1.4   0.00 -0.1    1.4  -7.14    0.0        -7.14   1.4   1.3     1.30     -7.14
3   3 -0.1    1.1  -9.09  0.3    0.9  33.33   -0.2        42.42   1.0   1.2     1.20     20.00
4   4 -0.5    1.6 -31.25  0.3    1.2  25.00   -0.4        56.25   1.1   1.5     1.50     36.36
5   5  0.6    1.8  33.33  0.7    1.3  53.85   -0.5        20.51   2.4   2.0     2.00    -16.67
6   6  0.5    2.8  17.86  0.0    2.3   0.00   -0.5       -17.86   3.3   2.3     2.30    -30.30
7   7  1.4    3.7  37.84  1.2    2.3  52.17   -1.4        14.34   5.1   3.5     3.50    -31.37
8   8  0.2    1.1  18.18  0.5    1.0  50.00   -0.1        31.82   1.3   1.5     1.46     15.38
9   9  0.8    2.1  38.10  0.8    1.8  44.44   -0.3         6.35   2.9   2.6     2.60    -10.34
10 10  1.2    2.8  42.86  0.3    2.9  10.34    0.1       -32.51   4.0   3.2     3.20    -20.00
11 11 -0.6    2.8 -21.43  1.3    1.6  81.25   -1.2       102.68   2.2   2.9     2.90     31.82
12 12 -0.3    1.6 -18.75 -0.2    1.8 -11.11    0.2         7.64   1.3   1.6     1.64     23.08

> # absolute differences in the FEF60 CHANGES at 0 min after exercise (Placebo-vit C), S&S Table II
> t.test(SchachterFEF60_0$PlCh, SchachterFEF60_0$VcCh, paired=TRUE, alternative='two.sided', mu=0.0,
conf.level=.95)
    Paired t-test
data:  SchachterFEF60_0$PlCh and SchachterFEF60_0$VcCh
t = -0.5606, df = 11, p-value = 0.5863
> # S&S reported t = 0.56 in Table II

> # absolute difference in FEF60 BASELINE levels (Placebo - vit C  = Base_D), S&S Table V
> t.test(SchachterFEF60_0$PlBase, SchachterFEF60_0$VcBase, paired=TRUE,  alternative='two.sided',
mu=0.0, conf.level=.95)
    Paired t-test
data:  SchachterFEF60_0$PlBase and SchachterFEF60_0$VcBase
t = 2.7437, df = 11, p-value = 0.01911
> # S&S reported t = 2.74 in Table V

> # Modification of the vitamin C effect by the placebo-day postexercise FEF60 change (P_D)

> LinearModel.31 <- lm(VC_Eff_FEF60 ~ 1       , data=SchachterFEF60_0)
> summary(LinearModel.31)
Coefficients:
            Estimate Std. Error t value Pr(>|t|)
(Intercept)    17.00      10.88   1.562    0.147
Residual standard error: 37.69 on 11 degrees of freedom

> LinearModel.32 <- lm(VC_Eff_FEF60 ~ 1 + P_D , data=SchachterFEF60_0)
> summary(LinearModel.32)
Coefficients:
            Estimate Std. Error t value Pr(>|t|)
(Intercept)  26.2467     9.7490   2.692   0.0226 *
P_D          -0.8978     0.3640  -2.466   0.0333 *
Residual standard error: 31.17 on 10 degrees of freedom
Multiple R-squared: 0.3782, Adjusted R-squared: 0.316
F-statistic: 6.082 on 1 and 10 DF,  p-value: 0.03332

> confint(LinearModel.32)
                2.5 %      97.5 %
(Intercept)  4.524443 47.96887820
P_D         -1.708873 -0.08667791

> # MODEL "32" ABOVE is shown in Fig 3C

> # This LR-test below shows that there is significant improvement in the model fit
> # when the placebo-day postexercise FEF60 change is added
> lrtest(LinearModel.31,LinearModel.32)
Likelihood ratio test
Model 1: VC_Eff_FEF60 ~ 1
Model 2: VC_Eff_FEF60 ~ 1 + P_D
  #Df  LogLik Df  Chisq Pr(>Chisq)
1   2 -60.058
2   3 -57.208  1 5.7017    0.01695 *

> # There is a significant difference in the pre-exercise FEF60 levels on the two days (t = 2.74),
see above
> # This model below tests whether the inclusion of Base_D influences the estimate for P_D
> LinearModel.33 <- lm(VC_Eff_FEF60 ~ 1 + P_D+ Base_D , data=SchachterFEF60_0)
> summary(LinearModel.33)
Coefficients:
            Estimate Std. Error t value Pr(>|t|)
(Intercept)  10.9407     9.4923   1.153  0.27877
P_D          -0.9464     0.2854  -3.317  0.00899 **
Base_D      -41.2360    15.2231  -2.709  0.02405 *
Residual standard error: 24.39 on 9 degrees of freedom
Multiple R-squared: 0.6575, Adjusted R-squared: 0.5813
F-statistic: 8.637 on 2 and 9 DF,  p-value: 0.008057
> # The estimate for P_D is NOT substantially changed
```

```
> # START Fig 3D : FEF60  -  0 minutes after exercise  ABSOLUTE LEVEL AFTER EXERCISE


> # Modification of the vitamin C effect by the placebo-day postexercise FEF60 level (PlEnd)

> LinearModel.41 <- lm(End_Ratio ~ 1         , data=SchachterFEF60_0)
> summary(LinearModel.41)
Coefficients:
            Estimate Std. Error t value Pr(>|t|)
(Intercept)   -1.397      7.272  -0.192    0.851
Residual standard error: 25.19 on 11 degrees of freedom

> LinearModel.42 <- lm(End_Ratio ~ 1 + PlEnd , data=SchachterFEF60_0)
> summary(LinearModel.42)
Coefficients:
            Estimate Std. Error t value Pr(>|t|)
(Intercept)   35.460     10.404   3.408  0.00668 **
PlEnd        -15.304      3.845  -3.980  0.00260 **
Residual standard error: 16.43 on 10 degrees of freedom
Multiple R-squared: 0.6131, Adjusted R-squared: 0.5744
F-statistic: 15.84 on 1 and 10 DF,  p-value: 0.002599

> confint(LinearModel.42)
                2.5 %    97.5 %
(Intercept)  12.27810 58.641412
PlEnd       -23.87077 -6.737327


> # MODEL "42" ABOVE is shown in Fig 3D

> # This LR-test below shows that there is significant improvement in the model fit
> # when the placebo-day postexercise FEF60 level is added
> lrtest(LinearModel.41,LinearModel.42)
Likelihood ratio test
Model 1: End_Ratio ~ 1
Model 2: End_Ratio ~ 1 + PlEnd
  #Df  LogLik Df  Chisq Pr(>Chisq)
1   2 -55.222
2   3 -49.525  1 11.394  0.0007368 ***


> # There is a significant difference in the pre-exercise FEF60 levels on the two days (t = 2.74),
see above
> # This model below tests whether the inclusion of Base_D changes the estimate for P_D
> LinearModel.43 <- lm(End_Ratio ~ 1 + PlEnd + Base_D, data=SchachterFEF60_0)
> summary(LinearModel.43)
Coefficients:
            Estimate Std. Error t value Pr(>|t|)
(Intercept)   36.644      9.598   3.818  0.00410 **
PlEnd        -18.692      4.073  -4.589  0.00131 **
Base_D       -18.194     10.846  -1.677  0.12776
Residual standard error: 15.12 on 9 degrees of freedom
Multiple R-squared: 0.7052, Adjusted R-squared: 0.6397
F-statistic: 10.77 on 2 and 9 DF,  p-value: 0.004099

> # The estimate for P_D is NOT substantially changed
```

Figs. 3E-F. Effect of vitamin C on the 0-min postexercise $FEF_{60(P)}$

In Figs. 3E and 3F the dash lines indicate the equality between vitamin C and placebo.
If vitamin C has no effect, the observations would be located randomly and symmetrically on both sides of the dash lines. The continuous lines indicate the regression lines.
The numbers identify the participants with the same numbers as used by S&S.

3E: Vitamin C effect on postexercise $FEF_{60(P)}$ change.
This figure shows the effect of vitamin C in percentage points (pp).
For example, on the placebo-day, participant #11 had a $FEF_{60(P)}$ change of -16.67%, and on the vitamin C day a $FEF_{60(P)}$ change of +88.89%, which gives the 105 pp improvement shown in Fig. 3E.
Slope of the linear regression model indicates that postexercise $FEF_{60(P)}$ change was decreased by 71% (95% CI: -3% to 145%, P = 0.06) on the vitamin C day compared with the change on the placebo day. Addition of the placebo-day postexercise $FEF_{60(P)}$ change to the model containing the intercept improved the model fit by χ^2(1 df) = 4.4 (P = 0.035).

3F: Vitamin C effect on postexercise $FEF_{60(P)}$ level.
This figure shows the effect of vitamin C in percentages.
For example, on the placebo-day, participant #11 had a postexercise $FEF_{60(P)}$ level of 2.5 L/s, and on the vitamin C day a postexercise $FEF_{60(P)}$ level of 3.4 L/s, which gives the 36% increase in the postexercise $FEF_{60(P)}$ level shown in Fig. 3F.
Slope of the linear regression model indicates that vitamin C had a significant effect on the postexercise $FEF_{60(P)}$ level (P = 0.02). Addition of the placebo-day postexercise $FEF_{60(P)}$ level to the model containing the intercept improved the model fit by χ^2(1 df) = 6.6 (P = 0.01).

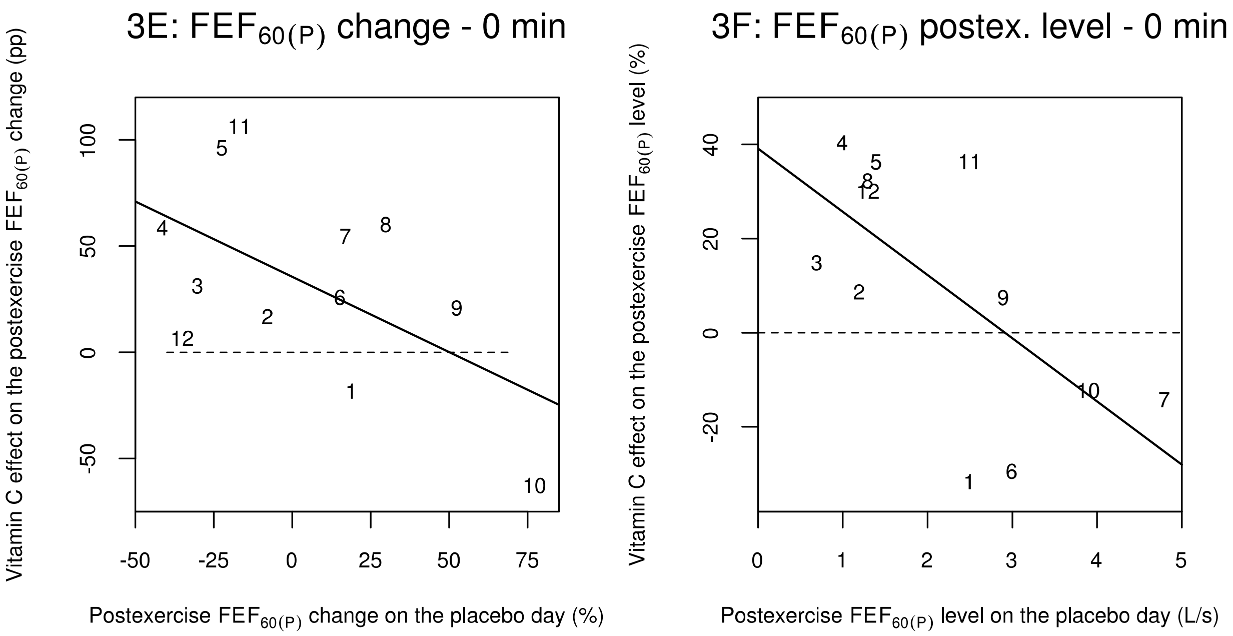

```
> # START Fig 3E : FEF60(P)  -  0 minutes after exercise   RELATIVE CHANGE
> SchachterFEF60_P_0
   NO PlCh PlBase    P_D VcCh VcBase   VC_D Base_D VC_Eff_FEF60P PlEnd VcEnd  End_Ratio End_Ratio_ed
1   1  0.4    2.1  19.05  0.0    1.7   0.00   -0.4        -19.05   2.5   1.7    -32.00       -32.00
2   2 -0.1    1.3  -7.69  0.1    1.2   8.33   -0.1         16.03   1.2   1.3      8.33         8.33
3   3 -0.3    1.0 -30.00  0.0    0.8   0.00   -0.2         30.00   0.7   0.8     14.29        14.29
4   4 -0.7    1.7 -41.18  0.2    1.2  16.67   -0.5         57.84   1.0   1.4     40.00        40.00
5   5 -0.4    1.8 -22.22  0.8    1.1  72.73   -0.7         94.95   1.4   1.9     35.71        35.71
6   6  0.4    2.6  15.38  0.6    1.5  40.00   -1.1         24.62   3.0   2.1    -30.00       -30.00
7   7  0.7    4.1  17.07  1.7    2.4  70.83   -1.7         53.76   4.8   4.1    -14.58       -14.58
8   8  0.3    1.0  30.00  0.8    0.9  88.89   -0.1         58.89   1.3   1.7     30.77        31.77
9   9  1.0    1.9  52.63  1.3    1.8  72.22   -0.1         19.59   2.9   3.1      6.90         6.90
10 10  1.7    2.2  77.27  0.4    3.0  13.33    0.8        -63.94   3.9   3.4    -12.82       -12.82
11 11 -0.5    3.0 -16.67  1.6    1.8  88.89   -1.2        105.56   2.5   3.4     36.00        36.00
12 12 -0.7    2.0 -35.00 -0.7    2.4 -29.17    0.4          5.83   1.3   1.7     30.77        29.77

> # absolute difference in FEF60(P) CHANGE at 0 min after exercise, S&S Table II
> t.test(SchachterFEF60_P_0$PlCh, SchachterFEF60_P_0$VcCh,paired=TRUE, alternative='two.sided',
mu=0.0, conf.level=.95)
    Paired t-test
data:  SchachterFEF60_P_0$PlCh and SchachterFEF60_P_0$VcCh
t = -1.6992, df = 11, p-value = 0.1173
> # S&S reported t = 1.66 in Table II

> # absolute difference in FEF60(P) BASELINE levels (BASE_D), S&S Table V
> t.test(SchachterFEF60_P_0$PlBase, SchachterFEF60_P_0$VcBase, paired=TRUE, alternative='two.sided',
mu=0.0, conf.level=.95)
    Paired t-test
data:  SchachterFEF60_P_0$PlBase and SchachterFEF60_P_0$VcBase
t = 2.0361, df = 11, p-value = 0.06655
> # S&S reported t = 2.04 in Table V

> # Modification of the vitamin C effect by the placebo-day postexercise FEF60(P) CHANGE (P_D)

> LinearModel.51 <- lm(VC_Eff_FEF60P ~ 1       , data=SchachterFEF60_P_0)
> summary(LinearModel.51)
Coefficients:
            Estimate Std. Error t value Pr(>|t|)
(Intercept)    32.01      13.54   2.364   0.0376 *
Residual standard error: 46.91 on 11 degrees of freedom

> LinearModel.52 <- lm(VC_Eff_FEF60P ~ 1 + P_D , data=SchachterFEF60_P_0)
> summary(LinearModel.52)
Coefficients:
            Estimate Std. Error t value Pr(>|t|)
(Intercept)  35.4724    11.9127   2.978   0.0139 *
P_D          -0.7092     0.3349  -2.118   0.0603 .
Residual standard error: 40.88 on 10 degrees of freedom
Multiple R-squared: 0.3096,	Adjusted R-squared: 0.2406
F-statistic: 4.485 on 1 and 10 DF,  p-value: 0.06026

> confint(LinearModel.52)
                2.5 %      97.5 %
(Intercept)  8.929384 62.01551266
P_D         -1.455447  0.03698215

> # MODEL "52" ABOVE is shown in Fig 3E in this Additional file 2

> # This LR-test below shows that there is significant improvement in the model fit
> # when the placebo-day postexercise FEF60(P) change is added
> lrtest(LinearModel.51,LinearModel.52)
Likelihood ratio test

Model 1: VC_Eff_FEF60P ~ 1
Model 2: VC_Eff_FEF60P ~ 1 + P_D
  #Df  LogLik Df  Chisq Pr(>Chisq)
1   2 -62.683
2   3 -60.460  1 4.4461    0.03498 *

# There is a marginally significant difference in the pre-exercise FEF60P levels
on the two days (t = 2.04), see above
> LinearModel.53 <- lm(VC_Eff_FEF60P ~ 1 + P_D + Base_D, data=SchachterFEF60_P_0)
> summary(LinearModel.53)
Coefficients:
            Estimate Std. Error t value Pr(>|t|)
(Intercept)  19.0066    11.3908   1.669   0.1295
P_D          -0.5229     0.2759  -1.895   0.0906 .
Base_D       38.0947    14.6144   2.607   0.0284 *
Residual standard error: 32.52 on 9 degrees of freedom
Multiple R-squared: 0.6066,	Adjusted R-squared: 0.5192
F-statistic: 6.939 on 2 and 9 DF,  p-value: 0.01502

# Thus the relation is nonsignificant when the baseline difference is added.
```

> # START Fig 3F : FEF60(P) - 0 minutes after exercise ABSOLUTE LEVEL AFTER EXERCISE

> # Modification of the vitamin C effect by the placebo-day postexercise FEF60(P) level (PlEnd)

```
> LinearModel.61 <- lm(End_Ratio ~ 1         , data=SchachterFEF60_P_0)
> summary(LinearModel.41)
Coefficients:
            Estimate Std. Error t value Pr(>|t|)
(Intercept)   -1.397      7.272  -0.192    0.851
Residual standard error: 25.19 on 11 degrees of freedom

> LinearModel.62 <- lm(End_Ratio ~ 1 + PlEnd , data=SchachterFEF60_P_0)
> summary(LinearModel.62)
Coefficients:
            Estimate Std. Error t value Pr(>|t|)
(Intercept)   39.083     12.496   3.128   0.0107 *
PlEnd        -13.420      4.951  -2.710   0.0219 *
Residual standard error: 20.95 on 10 degrees of freedom
Multiple R-squared: 0.4235, Adjusted R-squared: 0.3658
F-statistic: 7.346 on 1 and 10 DF,  p-value: 0.02192

> confint(LinearModel.62)
                2.5 %    97.5 %
(Intercept)  11.24014 66.925231
PlEnd       -24.45205 -2.387365
```

> # MODEL "62" ABOVE is shown in Fig 3F in this Additional file 2

> # This LR-test below shows that there is significant improvement in the model fit
> # when the placebo-day postexercise FEF60(P) level is added

```
> lrtest(LinearModel.61,LinearModel.62)
Likelihood ratio test
Model 1: End_Ratio ~ 1
Model 2: End_Ratio ~ 1 + PlEnd
  #Df  LogLik Df  Chisq Pr(>Chisq)
1   2 -55.746
2   3 -52.441  1 6.6091    0.01015 *
```

> # There is a marginally nonsignificant difference in the pre-exercise FEF60(P) levels on the two days (t = 2.04), see above
> # This model below tests whether the inclusion of Base_D changes the estimate for P_D
> LinearModel.63 <- lm(End_Ratio ~ 1 + PlEnd + Base_D, data=SchachterFEF60_P_0)
> summary(LinearModel.63)

```
Coefficients:
            Estimate Std. Error t value Pr(>|t|)
(Intercept)   39.007     13.057   2.987   0.0153 *
PlEnd        -14.129      5.468  -2.584   0.0295 *
Base_D        -4.024     10.043  -0.401   0.6980
Residual standard error: 21.89 on 9 degrees of freedom
Multiple R-squared: 0.4336, Adjusted R-squared: 0.3077
F-statistic: 3.445 on 2 and 9 DF,  p-value: 0.07746
```

> # The estimate for P_D is NOT substantially changed

Scatter plots of the pulmonary outcomes on vitamin C and placebo days – 0 min

The following figures show the scatter plots for the same variables as Figs. 3A to 3F on the vitamin C and placebo days, in the same order.
The dash line indicates the identity between vitamin C and placebo. If vitamin C did not differ from the placebo, the data points would be located symmetrically on both sides of the dash line.

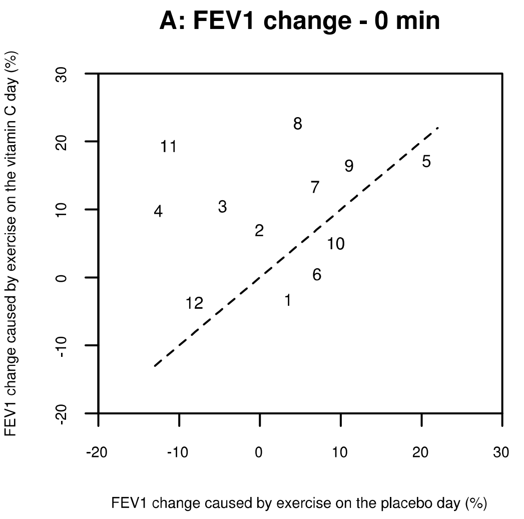

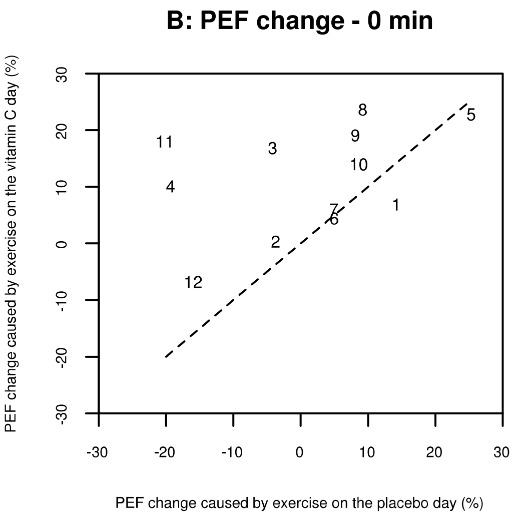

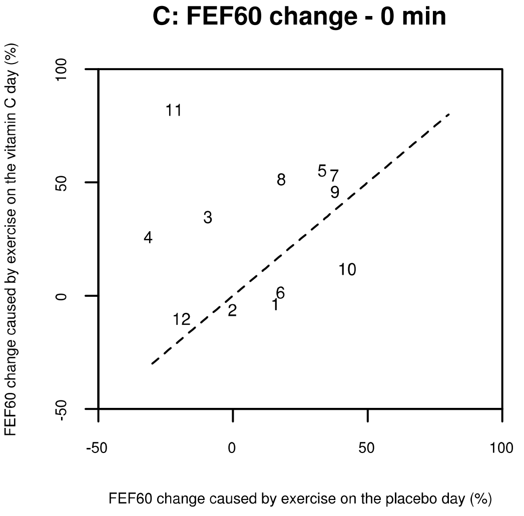

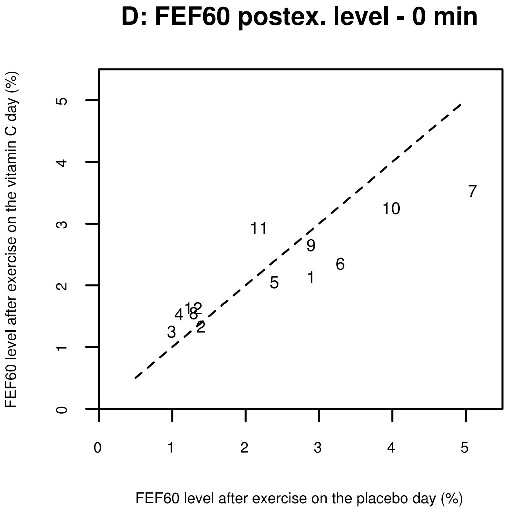

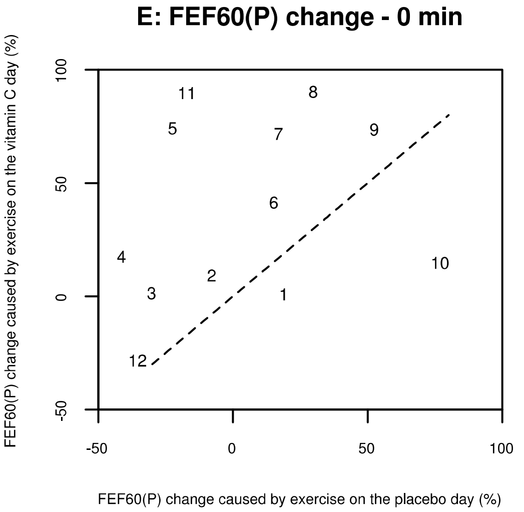

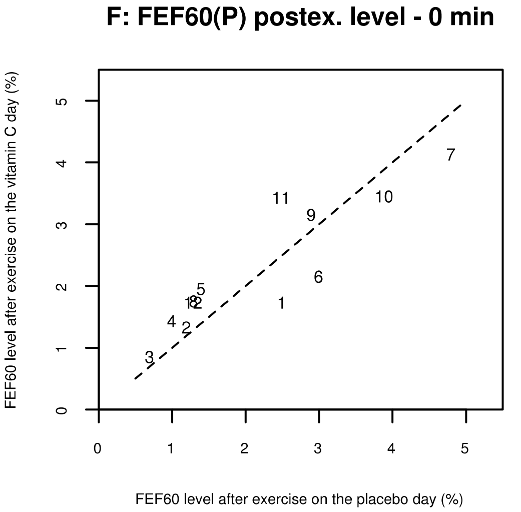

Supplement: Supplementary file 2 — Additional file 2: Methods for Figure 3 (0 min data) and Figures 3 E and 3 F. (PDF 236 KB) [file 13223_2014_588_MOESM2_ESM.pdf]
